# Global expansion of marine protected areas and the redistribution of fishing effort

**DOI:** 10.1073/pnas.2400592121

**Published:** 2024-07-09

**Authors:** Gavin McDonald, Jennifer Bone, Christopher Costello, Gabriel Englander, Jennifer Raynor

**Affiliations:** ^a^Marine Science Institute, University of California, Santa Barbara, CA 93106; ^b^Bren School of Environmental Science and Management, University of California, Santa Barbara, CA 93106; ^c^Environmental Markets Lab, University of California, Santa Barbara, CA 93106; ^d^Development Research Group, World Bank, Washington, DC 20433; ^e^Department of Forest and Wildlife Ecology, University of Wisconsin-Madison, Madison, WI 53706

**Keywords:** marine protected areas, conservation, fishing effort, predictive machine learning

## Abstract

The expansion of marine protected areas (MPAs) is a core focus of global conservation efforts, with the “30x30” initiative to protect 30% of the ocean by 2030 serving as a prominent example of this trend. We consider a series of proposed MPA network expansions of various sizes, and we forecast the impact this increase in protection would have on global patterns of fishing effort. We do so by building a predictive machine learning model trained on a global dataset of satellite-based fishing vessel monitoring data, current MPA locations, and spatiotemporal environmental, geographic, political, and economic features. We then use this model to predict future fishing effort under various MPA expansion scenarios compared to a business-as-usual counterfactual scenario that includes no new MPAs. The difference between these scenarios represents the predicted change in fishing effort associated with MPA expansion. We find that regardless of the MPA network objectives or size, fishing effort would decrease inside the MPAs, though by much less than 100%. Moreover, we find that the reduction in fishing effort inside MPAs does not simply redistribute outside—rather, fishing effort outside MPAs would also decline. The overall magnitude of the predicted decrease in global fishing effort principally depends on where networks are placed in relation to existing fishing effort. MPA expansion will lead to a global redistribution of fishing effort that should be accounted for in network design, implementation, and impact evaluation.

## Significance

The ability of marine protected areas (MPAs) to safeguard global biodiversity hinges on where and how much fishing effort occurs. Yet, MPA evaluations often simply assume that fishing effort inside new MPAs either disappears or moves elsewhere. We find that neither assumption is true. We use machine learning to understand how fishing fleets responded to past MPAs and to forecast the effects of future MPAs. We show that fishing effort decreases both inside and outside new MPAs. This finding suggests that MPAs have complex effects on fishing, which could be occurring through changes in fish stocks, costs, and/or profitability. As such, predicting and measuring the effect of MPAs on fishing effort is a critical part of marine spatial planning.

The expansion of marine protected areas (MPAs) is a crucial part of global conservation efforts (1). The “30x30” initiative, for example, aims to protect at least 30% of the world’s oceans by 2030 through a combination of fully protected areas (no extractive activities allowed) and partially protected areas (some activities remain permitted) (2, 3). Currently, fully protected MPAs cover less than 3% of the world’s oceans, but this is expected to increase (4, 5). As fully protected MPAs expand, it is crucial to understand their impact on global fishing effort. The creation of fully protected MPAs will shift the location and intensity of industrial fishing effort—as fishers move out of newly created MPAs, increased congestion and altered economic opportunities from fishing could lead to a cascade of redistribution that ripples from proximate to far-flung areas (6). But where and how much fishing effort will move remains an unanswered question.

The effectiveness and longevity of fully protected MPA expansion depends on how fishing effort responds. If fishing effort simply moves elsewhere, it could increase fishing intensity and threaten biodiversity outside of MPAs, possibly even reversing the presumed biodiversity benefits of protection (7). However, if fishing effort decreases (e.g., due to increased competition and reduced profitability), it could protect biodiversity (8) but potentially harm the economies of fishery-dependent nations and the feasibility of long-term protection commitments.

For example, two of the largest fully protected MPAs ever created, Phoenix Islands Protected Area and Palau National Marine Sanctuary, were recently reopened to fishing because of their perceived negative economic effects. In fact, protected area downgrading, downsizing, and degazettement of MPAs has been observed in dozens of MPAs around the world, with commercial fishing interests being one of the driving factors (9). Thus, understanding how fishing effort will redistribute must be a central component of marine spatial planning.

Previous research has measured the effect of individual MPAs on fishing responses, but typically for a single fleet in a limited area; none of the existing methods can capture the effects of global interventions affecting all fishing fleets. Simulation methods developed in the fisheries literature (10) and location choice models developed in the economics literature (6, 11–14) are helpful for understanding the structure of individual behavior, but are unlikely to apply when considering complex interactions between multiple fleets at the global scale. They also often require detailed vessel-level data, which are rarely available globally even with modern satellite tracking. Causal inference methods have been effective in examining regional effects of individual marine protection policies (15–18), but these methods require an unaffected control group, which by definition does not exist for a policy that induces global effects. As a result, simulations of large-scale MPA expansions have relied on heuristic assumptions of fisher responses, such as assuming no change outside MPAs or a uniform reallocation of effort from within MPAs to areas outside (19–23). This approach provides an “all else equal” reference point, but it is an assumed scenario, not an empirically driven response, so does not capture the true net effect of the policy.

**Fig. 1.**
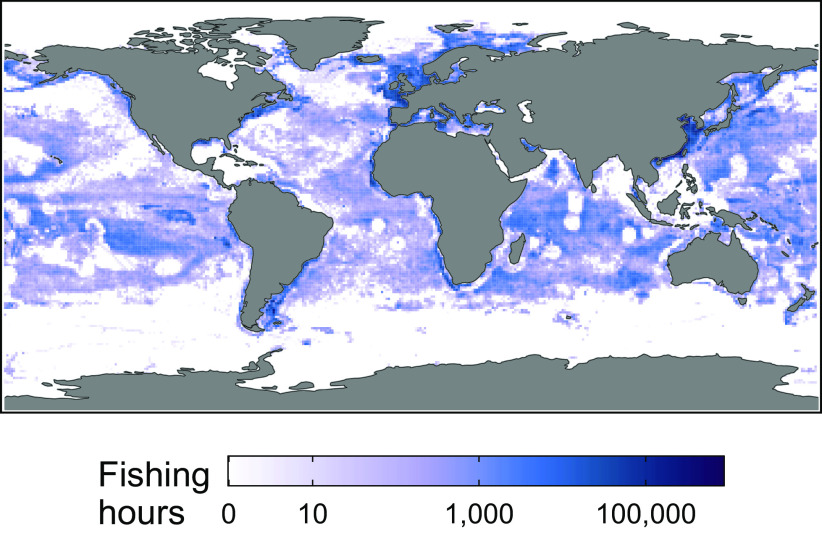
Map of observed fishing effort (hours) in 2021, shown using a log10 scale for visualization purposes. Pixels have a 1x1 degree geographic coordinate resolution, the spatial unit of our analysis.

We develop the first data-driven, predictive behavioral model of global fishing effort response following large-scale spatial closures. We begin by compiling a global dataset of fishing effort for all industrial fishing vessels that used Automatic Identification System (AIS) transponders between 2016 and 2021 (24), which is our outcome variable (Fig. 1). We then generate 42 model features that include spatial and temporal information on the geographic distribution of fully protected MPAs, environmental and economic conditions, and geographic and governance characteristics; we also assess AIS reception quality, which can affect apparent fishing effort from AIS transponders (see *Materials and Methods* for a complete list of model features). In order to promote computational tractability, we aggregate all data to a 1x1 degree pixel level annually; however, the model can be implemented, in principle, at any geographic resolution. Next, we train a series of two-stage hurdle random forest models to predict fishing effort one, two, and three years in the future: The first stage predicts whether any fishing occurs in a pixel, and the second stage predicts the intensity of fishing if it occurred. We tune the model hyperparameters using cross-validation (CV) with time-based folds, and we quantify out-of-sample performance over both time and space. Finally, we use the trained models to predict: i) a business-as-usual (BAU) counterfactual scenario, which represents future fishing effort if no new fully protected MPAs are implemented and ii) MPA expansion scenarios, which represent future fishing effort as fully protected MPA coverage incrementally increases from current levels. The difference between each MPA expansion scenario and the BAU counterfactual scenario represents the predicted change in fishing effort as a result of the MPA expansion. We focus our analysis on the potential impacts of fully protected MPAs, although in practice the levels of protection afforded by MPA expansion will vary across specific policies and regions. For example, under 30x30 the European Union has proposed to afford some level of protection to 30% of its waters, while fully protecting only 10% (25).

**Fig. 2.**
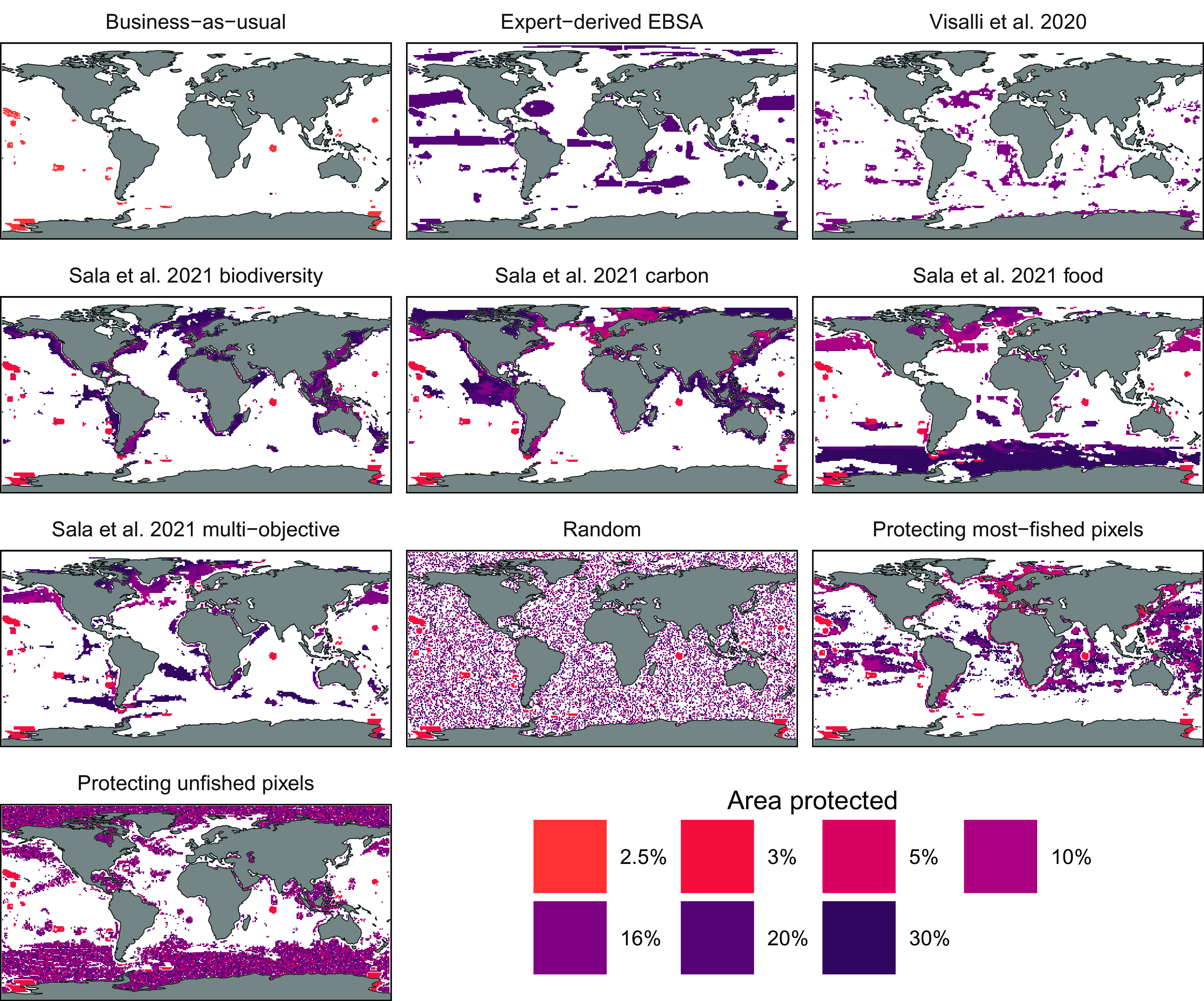
Maps of business-as-usual (BAU) network and hypothetical global MPA networks used in our simulations. The fill color of the global MPA network maps is by the global area coverage size, and only pixels that are fully enclosed in MPAs are colored. The BAU scenario holds fixed the existing fully protected MPA coverage as of the end of 2020 (2.5% of ocean area). Since the Sala et al. 2021 network scenarios, protecting most-fished pixel scenario, and random, unfished, and most-fished scenarios each protect pixels in descending order of priority, the network for each area protected size (3%, 5%, 10%, 16%, 20%, and 30%) is inclusive of all pixels in smaller coverage sizes. The Visalli et al. 2020 and expert-derived EBSA scenarios are each only available for a single coverage size (16% and 20%). Pixels have a 1x1 degree geographic coordinate resolution, the spatial unit of our analysis.

**Fig. 3.**
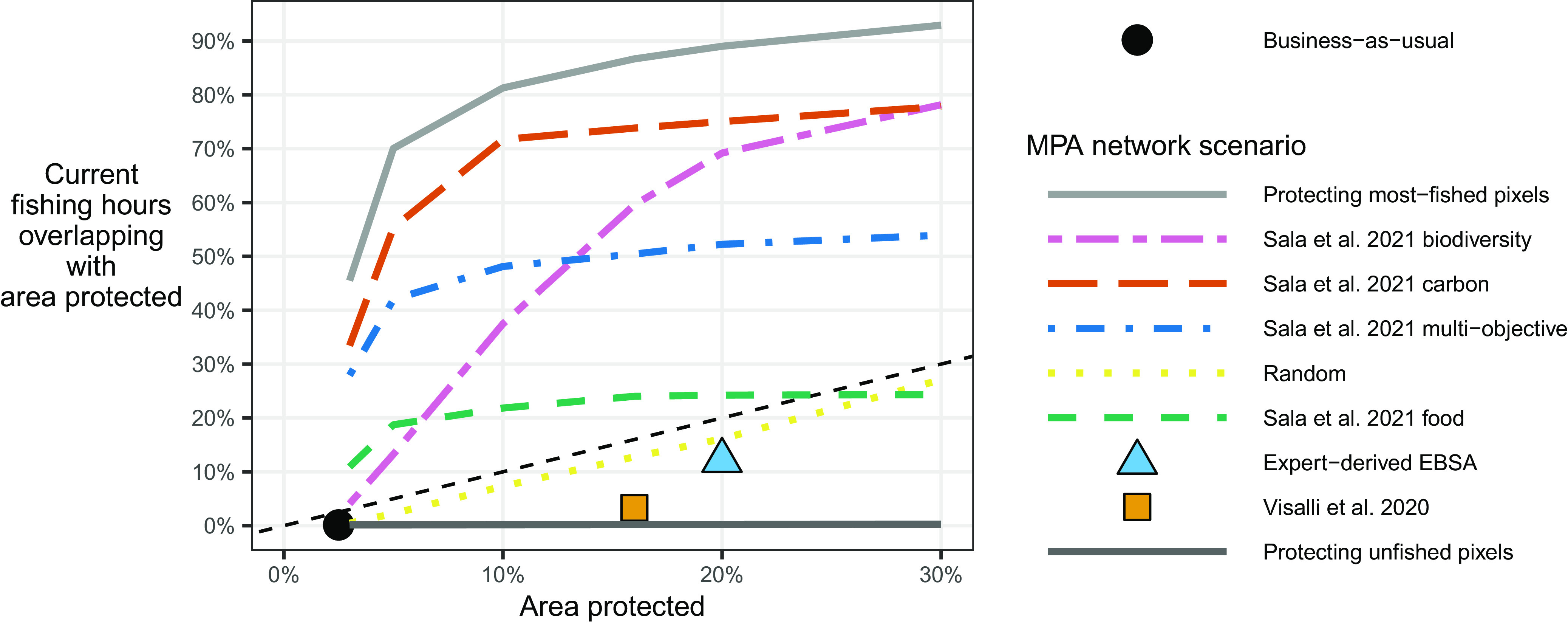
Percent of global fishing effort (hours) that spatially occurs within pixels that would be protected by hypothetical MPA networks versus percent of global ocean area protected, colored by MPA scenario. Colors differentiate the various hypothetical MPA networks. Linetypes are used to further differentiate networks that can have various levels of protection, while shapes are used to differentiate networks that only have a single level of protection. The scenarios are listed in the legend in the same order as they appear in the figure at the level of their largest area protected.

In forecasting the effects of future fully protected MPAs, we do not favor or limit our analysis to any particular MPA network proposal. Rather, we explore how a suite of alternative proposed MPA networks would each affect fishing effort. We include a number of networks that are the outputs of global prioritization analyses. These are a network that focuses on areas beyond national jurisdiction in the high seas (26), and a suite of networks that prioritize either biodiversity protection, carbon sequestration, food provision, or multiple objectives (20) (Fig. 2). We also consider an expert-designed bottom–up network of Ecologically or Biologically Significant Marine Areas (EBSAs) proposed by the Convention on Biological Diversity (27). We finally evaluate a set of networks that randomly protect pixels to achieve certain area-based targets, as well as networks that protect either the currently most-fished areas or the areas that are currently not fished. Importantly, each network differs in its overlap with current fishing effort (Fig. 1); for the same ocean coverage percentage, some networks would place MPAs in regions with significantly higher current fishing activity than others (Fig. 3). Comparing results across MPA networks therefore allows us to explore not only the potential impact of fully protecting, for example, 15% of the ocean, but also whether it matters which 15% of the ocean is protected.

We make three distinct scientific contributions. First, our results contribute to a timely, international policy debate on the impacts of large-scale MPA expansion and the best implementation strategies. While previous work has modeled the potential impacts of large-scale expansion of terrestrial protected areas on land (28), no such similar work has modeled the potential impacts of marine protected areas in the ocean. Second, we develop a machine learning technique for predicting global changes in fishing effort as a result of MPA implementation that is tractable, flexible, and data-driven. Machine learning lets us move beyond rigid assumptions about the structure of complex economic and ecological interactions because the algorithm allows for nonlinear, data-driven relationships (29). We also use machine learning to build a plausible BAU counterfactual, which allows for inference when there is no unaffected control group (30). Finally, our model can provide a decision-support tool for local marine managers to predict the effects of future spatial closures (fully protected MPAs or other spatially explicit fishing prohibitions). By providing more clarity on potential fishing effort outcomes, our model can help mangers reduce the probability of downgrading, downsizing, and degazettement of future MPAs, thus decreasing the uncertainty and regulatory burden associated with MPA expansion. Importantly, our model is general enough that it could also predict fishing redistribution from other spatiotemporal changes such as climate change.

## Results

How well can a globally tuned machine learning model actually predict fishing? An important first step in validation is to test the predictions of our trained models against out-of-sample fishing effort data. We find that the model performs well in these out-of-sample tests and is sufficient for our purpose of predicting future fishing effort under the expansion of fully protected MPAs. To test the model’s ability to predict future fishing effort, we perform a temporal out-of-sample test using a model trained on early years of the dataset and tested on held-out later years of the dataset. Using this test, the receiver operating characteristic (ROC) area-under-the-curve in the first stage prediction is approximately 0.97 and the F1 score is approximately 0.91 (*SI Appendix*, Fig. S4*A* and Table S1). In the second stage prediction of fishing intensity, the *R*^2^ is approximately 0.8 (*SI Appendix*, Fig. S4*B* and Table S1). Additionally, across performance metrics, there is little reduction in performance as we predict fishing effort additional years into the future. To test the model’s ability to predict both future fishing effort and in spatial areas that have never before seen fully protected MPAs, we perform a spatiotemporal out-of-sample test that uses a suite of models for each ocean trained on early years of the dataset and in other oceans, and tested on held-out later years of the dataset and in the ocean of interest. We do this leave-one-out test for each ocean, allowing us to see how well the model can predict fishing effort in spatial areas where the model has not seen any training data. Again, we find high performance for the spatiotemporal out-of-sample testing (*SI Appendix*, Fig. S5), indicating that the model can forecast across both time and space. Finally, we test our model’s performance against a series of simpler models, again using temporal out-of-sample testing. We find that our model outperforms these simpler models across all performance metrics (*SI Appendix*, Fig. S6). These out-of-sample evaluations give credence to our subsequent MPA network scenario predictions because the MPA network scenario predictions consider MPAs in future years and in locations that may not yet have MPAs.

**Fig. 4.**
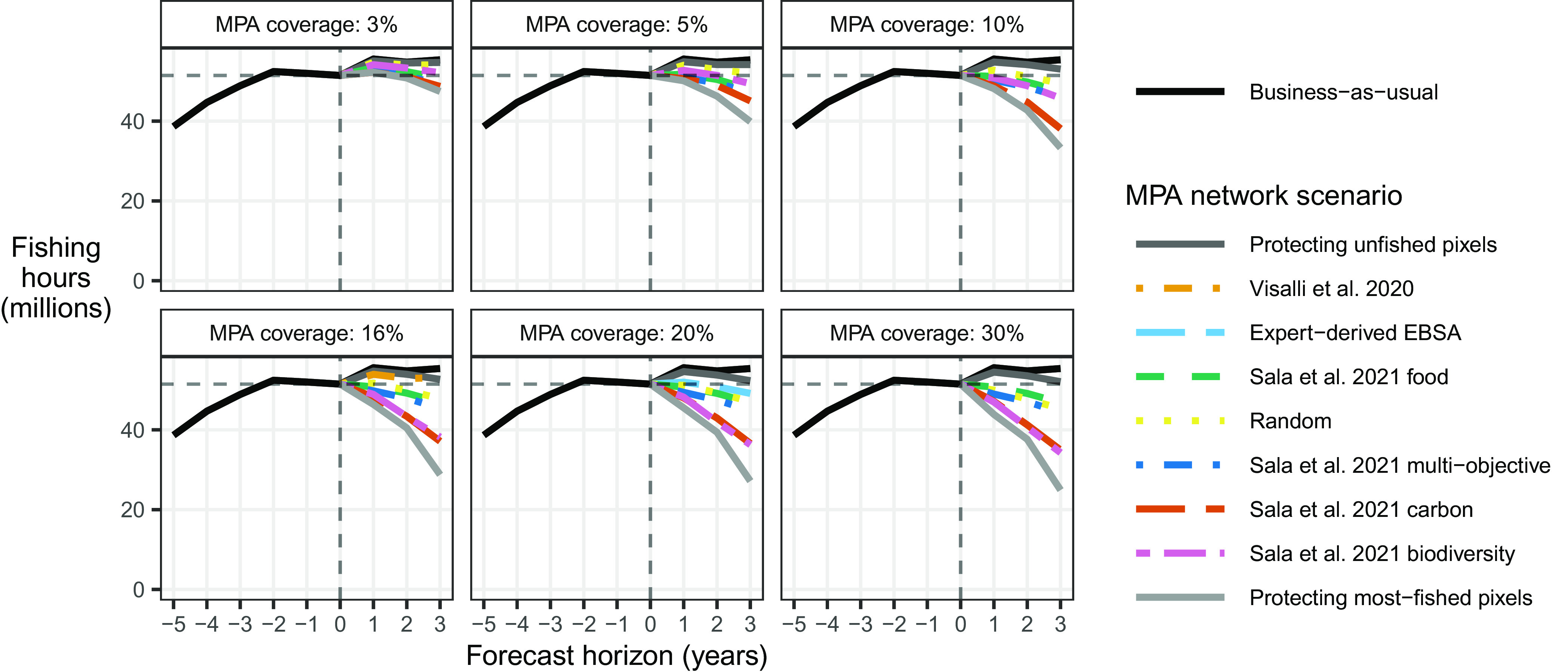
Total observed global fishing effort (hours) (where forecast horizons −5 to 0 correspond to observed data from years 2016 to 2021), and total predicted global fishing effort in the business-as-usual (BAU) scenario and all MPA scenarios for the three predicted forecast horizons. A vertical dashed line is shown at 0 y (such that the line to the *Left* represents observed data, and the lines to the *Right* represent predictions). A horizontal dashed line is shown at the level of currently observed fishing effort in the last year of historically observed data. Each panel represents global MPA networks that are sized for a given percentage coverage. Colors and linetypes differentiate the various hypothetical MPA networks and the BAU scenario. The MPA network scenarios are listed in the legend in the same order as they appear in the figure at a forecast horizon of 3 y and their largest MPA coverage.

In the absence of any new MPAs, our BAU counterfactual scenario predicts greater total global fishing effort in the future compared to currently observed levels (Fig. 4). Under any of the hypothetical MPA expansion scenarios we consider, our model predicts that total future global fishing effort will invariably be lower than the BAU counterfactual scenario of no new MPAs. The extent of this decrease depends on the MPA network and the percentage of ocean area it encompasses. For some scenarios such as protecting unfished pixels, “Visalli et al. 2020,” “Expert-derived EBSA,” “Random,” and for MPA coverage levels of 5% to 20%, we find that predicted total fishing effort may be roughly equal to or even above the current levels we see today. However, for other scenarios, and all scenarios with 30% protection (other than protecting unfished pixels), we find that total global fishing effort would be both lower than the currently observed level and lower than the predicted future level under business-as-usual.

**Fig. 5.**
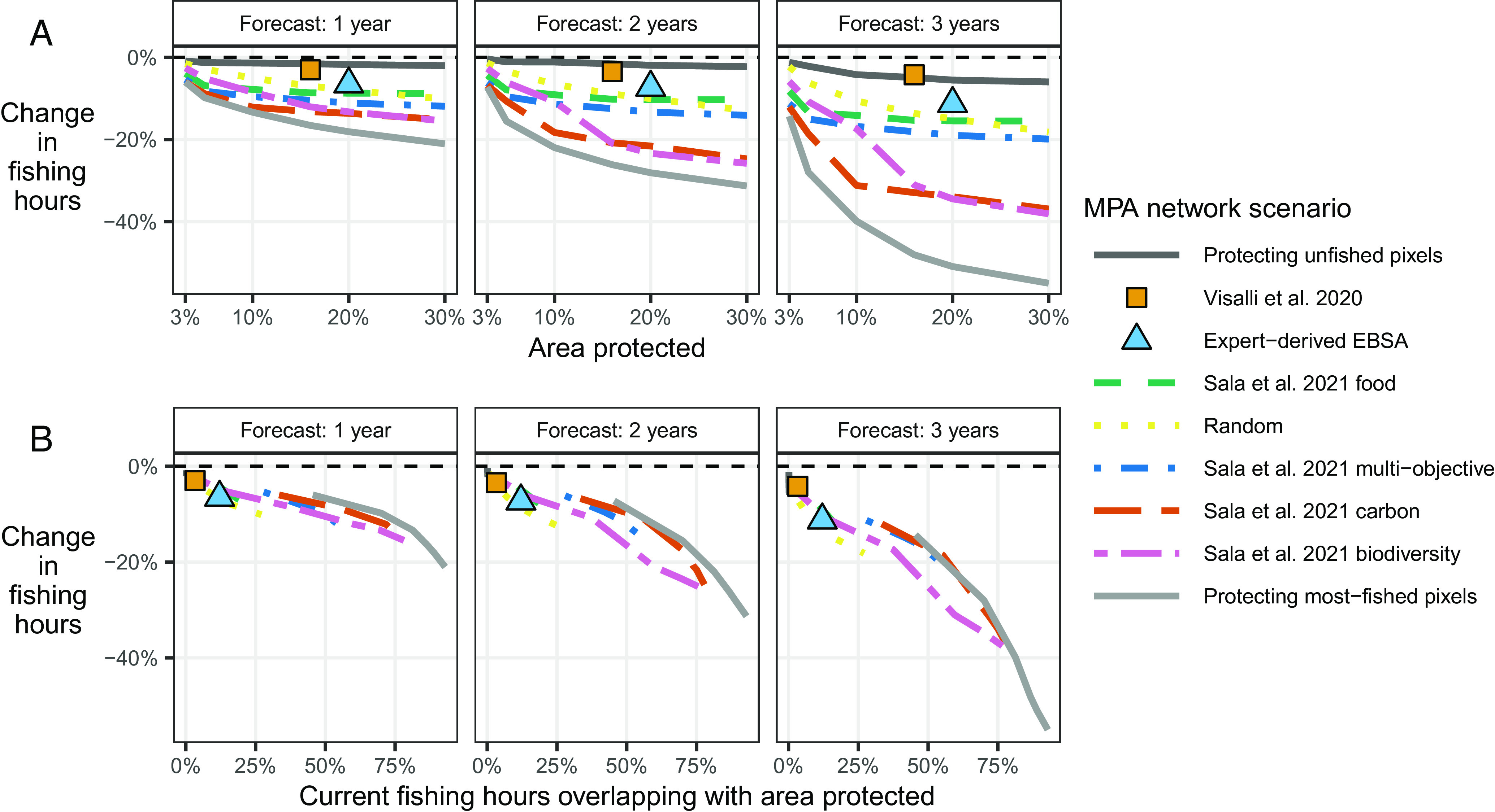
Predicted aggregate percentage changes in global fishing hours from expanding MPAs. The y-axis shows the relative difference in fishing hours between each MPA network scenario and the business-as-usual counterfactual scenario, with values aggregated globally. (*A*) presents the relationship between the percentage change in fishing hours and the percentage of ocean area covered by each hypothetical MPA network, with panels for each of the three forecast horizon years. (*B*) presents the relationship between the percentage change in fishing hours and the percentage of current global fishing hours occurring in areas that would be covered by each hypothetical MPA network, with panels for each of the three forecast horizon years. In all panels, colors differentiate the various hypothetical MPA networks. Linetypes are used to further differentiate networks that can have various levels of protection, while shapes are used to differentiate networks that only have a single level of protection. The MPA network scenarios are listed in the legend in the same order as they appear in the *Top Right* panel and their largest MPA coverage.

Intuitively, the decrease in future total fishing from MPA expansion is smallest under the scenarios that extend protection to areas that are currently unfished (−0.4% to −6%, dark gray line in Fig. 5*A*). Scenarios that extend protection to areas that are currently most fished would lead to the largest decrease in total fishing effort (−6% to −55%, light gray line). These two scenarios are not meant to represent plausible real-world MPA networks; rather, they are intended to display a range of possible effects from the large-scale expansion of fully protected MPAs. Most of the actual proposed networks result in reductions in fishing effort of about 10% to 20%. Two exceptions are “Sala et al. 2021 carbon” and “Sala et al. 2021 biodiversity,” which have predicted declines of 37% and 38%, respectively, after three years and with full 30% area protection. For all scenarios, as the percentage of ocean area protected increases, we predict incrementally larger decreases in total fishing effort.

We have shown that across the range of proposed protection scenarios (i.e. excluding the “Most-fished,” “Unfished,” and “Random” scenarios), global fishing effort is likely to decrease, and the magnitude ranges from about −3% to −38%. What drives the magnitude of predicted decreases for different MPA networks? Regardless of the network’s objectives, how it was designed, the percentage of the ocean covered, or the forecast horizon, the key driver is the overlap of the proposed network with current fishing effort (Fig. 5*B*). As this percentage increases, we consistently predict larger decreases in global fishing effort. For instance, the proposed scenario that results in the smallest predicted decrease covers only 4% of current global fishing hours. Similarly, the proposed scenario prompting the largest predicted decrease covers the greatest percentage of current global fishing hours (78%). This pattern implies that the overlap between current fishing effort and new MPAs will play a crucial role in determining the impacts of future MPA expansion.

**Fig. 6.**
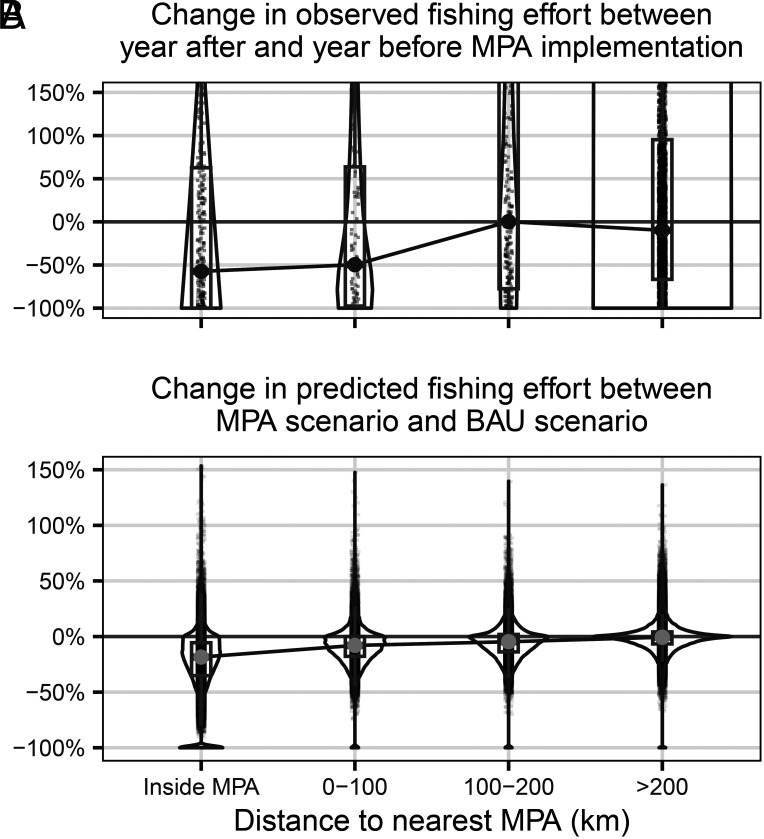
(*A*) Observed historic pixel-level changes in fishing hours between one-year after MPA implementation and one-year before MPA implementation, for fully protected MPAs implemented after 2016 and before 2021. Pixels are bucketed into different distance bins to the nearest MPA. (*B*) Predicted pixel-level changes in fishing hours from expanding MPAs, relative to business-as-usual scenario, with pixels grouped into different distance categories to the nearest MPA. Each point represents the pixel-level results for a different hypothetical MPA scenario, coverage size, and forecast horizon. For both (*A* and *B*), the points are jittered to avoid visual overlap. Boxplots and violin plots show distributions for each bin. The boxplots for each bin show the median, 25th percentile, and 75th percentile. A line connects the median values for each distance bin. The y-axis is limited to −100% to 150%, although some outlier values extend beyond 150%.

Crucially, these reductions in aggregate fishing effort arise both inside and outside the new MPAs. Aggregate fishing effort inside the MPAs diminishes under all network scenarios; however, it never drops to zero, even though these new MPAs ostensibly prohibit all commercial fishing (*SI Appendix*, Figs. S11 and S12). Aggregate fishing effort outside MPAs also decreases across all scenarios, which drives the majority of the global decline (*SI Appendix*, Figs. S11 and S12). This effect is most prominent in locations closer to MPA boundaries. Pixels located fully inside new MPAs see the largest median decrease in fishing effort (−18%), and this median decrease becomes smaller and approaches zero as the distance increases from the MPA boundary (Fig. 6*B*). This spatial dissipation is consistent with the before-versus-after changes we observe in the historical raw data following the implementation of real MPAs (with an observed median decrease of −57% for pixels fully inside new MPAs, and diminishing magnitude changes as the distance to the nearest MPA increases) (Fig. 6*A*).

How do our results compare to the conventional wisdom? The two most common assumptions employed in the previous literature are “full displacement” (where each unit of fishing effort covered by an MPA is displaced outside the MPA) and “complete exit” (where each unit of fishing effort covered by an MPA disappears) (19–23). Our results suggest that neither of these hypotheses is correct. Instead, we find that the large-scale expansion of fully protected MPAs is likely to trigger a redistribution of fishing effort, most prominently near new MPAs but with effects that also arise farther away, which in aggregate imply a global reduction in fishing. Furthermore, as fully protected MPAs cover larger fractions of current fishing activity, this reduction magnifies, underscoring the importance of carefully considering fishers’ adaptation to MPA expansion.

## Discussion

The ecological and economic consequences of large-scale MPA expansion will hinge to a large extent on how global fishing effort responds to increased protection, yet little is known about how this will unfold. Understanding the potential redistribution of fishing effort, and the subsequent changes in the aggregate quantity of global fishing, is an important first step. We developed an empirical global model to predict how fishing activity will respond to changes in fully protected MPA coverage. Importantly, our model is general enough that it could also be used to predict fishing changes in response to other oceanic changes, such as climate change or new fishing regulations. Across a wide range of hypothetical MPA networks, our key finding is clear: Aggregate global fishing is likely to decline, and the magnitude of this decline is largely driven by the amount of current fishing activity in the newly protected areas. This factor is more important than either the conservation objectives of the MPA network or even the total area it covers. Specifically, when new fully protected MPAs are placed in regions currently experiencing intense fishing activity, we predict the most substantial decreases in total fishing effort. While policymakers may choose to place MPAs in locations with limited fishing activity—for strategic reasons such as protecting critical habitats, or for political reasons such as protecting areas that are currently unfished or minimally fished—our results suggest that such placements would exert minimal impact on fishers’ decisions. In other words: which parts of the ocean are protected is more important in determining overall fishing effort than how much of the ocean is protected.

Our results show that new fully protected MPAs will lead to a global redistribution of fishing effort, but we also find that other factors play an important role in determining fishing effort. While model features based on MPAs make important contributions toward predictions of future fishing effort (*SI Appendix*, Figs. S7, S9, and S10), in aggregate they are less important than features like previous fishing effort and spatiotemporal environmental factors (*SI Appendix*, Fig. S8). Climate change, which may have impacts on these spatiotemporal environmental factors such as sea surface temperature and the El Niño-Southern Oscillation (31), could therefore play an important role in the redistribution of future fishing effort. In fact, changes in sea surface temperature are already playing a role in the redistribution of tuna catch in the Eastern Pacific Ocean (32).

Because our model is training on historical data, the validity of our predictions relies on the assumption that future fisher responses to MPAs will resemble past responses of fishers to MPAs. While our MPA expansion scenarios represent different degrees of extrapolation from past experience, it is reassuring that predictions across proposed MPA networks consistently align when assessed in relation to the percentage of current fishing activity they cover. Our predicted percentage changes inside and nearby MPAs are also consistent with the changes seen in the historically observed data for MPAs that were implemented after 2016 and before 2021 (Fig. 6).

Although our results consistently predict that large new networks of MPAs will lead to decreased global fishing effort, decreases in effort do not necessarily translate to decreases in catch, food provisioning, catch-per-unit-effort (CPUE), revenue, or profits. A recent review paper found 48 examples of fisheries benefits relating to MPAs across 25 countries (33). For example, an empirical analysis of the impacts from Papahānaumokuākea Marine National Monument found that CPUE increased in areas surrounding the MPA as a result of the MPA (34). Increased CPUE—as empirically shown in this example—combined with decreased effort—as predicted by our model—could lead to either increases or decreases in catch, depending on the relative magnitude of the changes of each. A study of Revillagigedo National Park in Mexico, the largest fully protected MPA yet to be implemented in North America, found that while the MPA reduced fishing effort, CPUE did not significantly change (35). In general, constant CPUE combined with decreased effort would lead to decreases in catch. Aside from potential fisheries impacts, benefits from MPAs relating to tourism have also been widely documented (33).

The impact of MPAs on catch and biomass will depend on current fishery status and other types of existing fisheries management institutions. In fisheries that are currently overfished (e.g., catch exceeds maximum sustainable level), standard fisheries surplus production models predict that reducing fishing effort will increase both catch and biomass (36). But in fisheries that are currently fished at or below sustainable levels, reducing fishing effort may reduce catch (while still increasing biomass).

MPAs are often not used in isolation but in the context of other types of fisheries management. As an alternative or complement to spatial protections, fisheries management has been shown to effectively increase biomass while simultaneously increasing catch and profits (36, 37). While our current analysis on the impact of global MPA expansion on fishing effort is an important first step, future research could use bioeconomic simulation modeling to explore how this predicted change in fishing effort would translate to changes in catch, CPUE, revenue, or profits.

While the decrease in fishing effort within the bounds of newly created MPAs aligns with expectations and historically observed patterns (38), our consistent prediction that fishing effort will also decrease outside of MPAs is more surprising. This is in contrast to the commonly discussed concept of “fishing-the-line,” in which fishing effort concentrates around the edges of newly created MPAs (39). Here, we hypothesize three potential mechanisms that could lead to our result. In practice, different mechanisms may be at play under different contexts, and multiple mechanisms may simultaneously occur. While it is outside the scope of this paper, future research could empirically examine which of these different explanations are most important and in which contexts.

First, when there is significant biological spillover, which refers to the movement of fish and other marine life from newly protected areas to the remaining fishing grounds, CPUE may actually increase in the remaining fishing grounds. This increase could allow vessels to reduce their fishing effort while maintaining catch levels (a decision-making strategy known as “satisficing” in the economics, psychology, and political science literatures). This phenomenon may also arise when catch is closely regulated outside the protected area.

A second possible explanation is that under certain circumstances, a new MPA may close off the most productive fishing grounds, leaving less productive areas open to fishing. If there is little or no biological spillover of the region’s target species, fishing in the remaining open areas may become less profitable. If this is the case and fishers use a “profit-maximizing” decision-making strategy, this reduced profitability could lead to a decrease in fishing effort outside the MPA.

A third possible explanation is that the fixed cost of traveling to distant waters could lead to a decrease in fishing outside large and distant MPAs. Consider a scenario where the MPA generates negligible biological spillovers for the target species and where the MPA covers a large fraction of a fleet’s fishing grounds. Some distant water fishing fleets that specialize in fishing this species and region may have historically incurred large fixed costs to transit from their home ports to the fishing grounds. If a large fraction of those historical grounds are closed to fishing by an MPA, fishing the remaining open area may become unprofitable, so the fleet may divest entirely from those fishing grounds, and we would observe a decrease both inside and outside the MPA. This fixed cost effect could even be compounded by displacement to less productive fishing grounds which have lower CPUE, the second mechanism discussed above.

Our discussion of all three mechanisms is speculative; we merely intend to offer potential interpretations of our results and motivate future research. In that spirit, we share the following real-world example regarding our third mechanism. In 2020 the Pacific island nation of Palau closed 80% of its Exclusive Economic Zone (EEZ) to fishing, and the Taiwanese fleet, which was the largest fleet fishing in Palau, is said to have left Palau entirely. Indeed, an inspection of global fishing effort reveals that this is exactly what happened. In the year following Palau’s MPA implementation, Taiwan’s fishing effort decreased by over 99% inside the new MPA and by 62% outside the MPA, for a total decrease of 97% (*SI Appendix*, Fig. S13). This finding is corroborated by a major Taiwanese-owned fishing company that stated that the reduced size of Palau’s fishing grounds caused by the MPA made it no longer financially viable to continue operating anywhere in Palau (40). While fixed costs may substantially reduce fishing by some fleets, other fleets may respond differently. For example, prior to 2020, Japan was the second-largest fleet operating in Palau. After the establishment of the MPA, Japanese fishing effort decreased by 95% inside the MPA, but actually increased outside the MPA by 155%, leading to a net increase in Japanese fishing in Palua’s waters of 0.2%. Since both fleets predominantly use drifting longlines in Palau and are therefore likely fishing for similar species that would have similar biological responses to the MPA, the different behavioral responses from these two fleets suggest that economics plays an important role in the spatial changes in fishing effort.

We next investigate whether this anecdote can also exemplify our main finding that large-scale expansion of fully protected MPAs is likely to reduce global fishing effort. We do so by following Palau’s “pre-MPA fishing fleet,” which consists of all vessels that fished in Palau’s EEZ prior to the 2020 MPA implementation, regardless of their flag. While some of these vessels reallocated fishing activity to regions outside Palau’s EEZ (*SI Appendix*, Fig. S14*B*), others ceased fishing entirely (*SI Appendix*, Fig. S14*A*). Between 2019 and 2021, the number of active vessels who fished anywhere in the world from Palau’s pre-MPA fishing fleet decreased in size from 202 to 159 vessels (−21%). The pre-MPA fishing fleet’s global fishing effort also decreased by 49% between 2019 and 2021. By comparison, global fishing effort across all vessels decreased by only 1.9% between 2019 and 2021.

Global reductions in fishing effort will occur across a range of habitats, which could have ecological benefits for both biodiversity and fish populations. We can gauge these benefits by overlaying the predicted spatial changes in fishing effort with regions of biological or ecological interest. There are many different ways to do this, so as one example, we overlay our spatial predictions with the spatial boundaries of Large Marine Ecosystems (LMEs) (41) (*SI Appendix*, Fig. S15). LMEs represent regions in continental coastal waters characterized by trophically dependent populations and higher primary productivity compared to open-water areas. They can thus be relevant for informing ecosystem-based management and MPA network design. Naturally, the specific changes in each LME will be contingent on the exact placement of new MPAs. Given that we predict the largest decreases in effort inside the boundaries of new MPAs, we can expect that fishing pressure will be reduced most in those habitats directly protected by new MPAs. A clear pattern emerges across the range of hypothetical networks we considered in our analysis: Regardless of the MPA network scenario, almost all LMEs will experience a decrease in fishing effort compared to business-as-usual. Similar to understanding how predicted changes in effort would translate to predicted changes in catch, making precise predictions for the resulting biological effects would require explicit modeling of population dynamics (42).

Since our analysis uses AIS-based vessel monitoring as the basis of our global dataset of observed fishing effort, it is important to acknowledge that not all fishing vessels use AIS, and thus our results are likely most relevant to those that do. AIS can be used to monitor most of the world’s large fishing vessels above 24 m in length (43); the dataset in our analysis represents fishing activity by over 110,000 fishing vessels between 2016 and 2021. However, most vessels below 24 m do not use AIS (43), such as many small-scale, artisanal, or subsistence fishing vessels. Our results should therefore be interpreted with caution when trying to understand how MPAs may impact fishing effort of small-scale vessels. Even for industrial fleets, a recent analysis using satellite imagery data found that many fishing vessels do not broadcast AIS (44). Future research could empirically examine how effort by these “dark” vessels respond to the implementation of MPAs.

Our findings, indicating a decrease in global fishing effort both inside and outside of MPAs regardless of the hypothetical network scenario, suggest that expanding fully protected MPA coverage would likely benefit fish populations. This could also benefit fishers who are operating in fisheries that are currently experiencing overfishing. However, fishers may bear an economic cost in fisheries that are currently being fished more conservatively. This divergence underscores an important challenge that international conservation initiatives like 30x30 confront: balancing ambitious conservation goals with the livelihoods of those who depend on the sea. Achieving such a balance is possible, and we are not arguing in this paper against MPA expansion or that MPAs will have a negative impact on fisheries. Future research could strive to furnish direct evidence regarding both the conservation impacts and economic fisheries impacts of proposed global ocean interventions. Such understanding will be essential for formulating policies that effectively protect marine ecosystems and economically benefit fisheries-dependent people.

## Materials and Methods

### Data Processing and Feature Engineering.

We build a global, spatial-temporal dataset of AIS fishing hours and 42 model features that predict the location and intensity of fishing effort. For each feature, we discuss the rationale for its inclusion in the model and data processing steps below. Table 1 summarizes the complete list of features, measurement units, variable types, spatial-temporal variation, and data sources. For all features, our observation unit is a 1x1 degree pixel-year, although our model specification can theoretically handle any spatial-temporal resolution.

**Table 1.** Data sources for all included model features, including the units, feature type, whether it varies spatially, whether it varies temporally, and the source name and reference citation

No.	Feature (units)	Type	Spatial	Temporal	Source	Ref.
MPA implementation						
1	Nearest MPA: distance (m)	Numeric	Yes	Yes	MPA Atlas, December 2020	(5)
2	Nearest MPA: years since designation (years)	Numeric	Yes	Yes	MPA Atlas, December 2020	(5)
3 to 5	Spatial coverage: fraction of pixel, first and second neighbor	Numeric	Yes	Yes	MPA Atlas, December 2020	(5)
6	Spatial coverage: inside, outside, or partial	Categorical	Yes	Yes	MPA Atlas, December 2020	(5)
7	Temporal coverage: fraction of year	Numeric	Yes	Yes	MPA Atlas, December 2020	(5)
8 to 11	Future coverage: inside or partial, 1 and 2 y leads	Boolean	Yes	Yes	MPA Atlas, December 2020	(5)
Environmental (mean and sd for each)						
12 to 15	Sea surface temperature and anomaly (°C)	Numeric	Yes	Yes	NOAA 0.25-deg Daily OI SST V2.1	(46)
16 and 17	Chlorophyll-A (mg/m^3^)	Numeric	Yes	Yes	Aqua MODIS Chl-a 4 km monthly	(47)
18 and 19	Wind speed (m/s)	Numeric	Yes	Yes	CCMP Wind Vector V2.1, 4x daily	(48)
20 and21	El Niño Southern Oscillation index	Numeric	No	Yes	NOAA ENSO 3.4 Index	(49)
22 and 23	Pacific Decadal Oscillation index	Numeric	No	Yes	NOAA PDO Index	(50)
Geographic						
24 and 25	Latitude and longitude (°)	Numeric	Yes	No	Global Fishing Watch	(24)
26	Shore: nearest distance (m)	Numeric	Yes	No	Global Fishing Watch	(24)
27	Seamount: nearest distance (m)	Numeric	Yes	No	Yesson et al. 2020	(51)
28	Bathymetry: depth (m)	Numeric	Yes	No	Global Fishing Watch	(24)
29	Ocean	Categorical	Yes	No	Marine Regions Global Oceans	(52)
30	Mesopelagic zone	Categorical	Yes	No	Biogeographic mesopelagic zones	(53)
Governance						
31	Exclusive Economic Zone: sovereign state	Categorical	Yes	No	Marine Regions V11	(54)
32	Exclusive Economic Zone: fraction of pixel coverage	Numeric	Yes	No	Marine Regions V11	(54)
33	Exclusive Economic Zone: nearest distance (m)	Numeric	Yes	No	Marine Regions V11	(54)
33	Exclusive Economic Zone: nearest sovereign state	Numeric	Yes	No	Marine Regions V11	(54)
34	World Bank Development Indicators region	Categorical	Yes	No	R “countrycode” package	(55)
35	Global Fishing Index governance capacity	Categorical	Yes	No	Global Fishing Index	(56)
Economic						
36	Distance from port (m)	Numeric	Yes	No	Global Fishing Watch	(24)
37 and 38	IFO 380 fuel price, mean and sd (USD/MT)	Numeric	No	Yes	Bunker Index	(57)
Technological						
39 and 40	AIS reception: Type A and Type B transponders (messages/day)	Numeric	Yes	No	Global Fishing Watch	(24)
Residual effects						
41	AIS fishing effort: 1-, 2-, or 3-y lag (log(h/m^2^))	Numeric	Yes	Yes	Global Fishing Watch	(24)
42	Year	Numeric	No	Yes	Global Fishing Watch	(24)

1. Outcome variable. Our outcome variable is AIS fishing effort (hours) from Global Fishing Watch (GFW) between 2016 and 2021 (24). We normalize the fishing effort in each pixel by the spatial area of each pixel (m^2^) in order to account for slightly different pixel sizes across the globe, depending on the latitude. We also log transform fishing effort because it is a highly skewed distribution. Our final measurement unit is therefore log(h/m^2^). After making predictions, we backtransform the predicted log fishing effort to obtain predicted level fishing effort (45), and then multiply this area-normalized effort by the pixel area to obtain absolute predicted fishing effort. We do not use earlier years of GFW data (2012 to 2015) because AIS coverage and quality were generally increasing during those years; these trends could cause confounding in the model because MPA expansion is also increasing over time.
2. MPA implementation. We delineate the spatial-temporal spread of MPAs globally. For each year, we determine the global network of fully protected MPAs that had been implemented during or before that year. The boundaries of MPAs come from a version of the MPA Atlas downloaded in December 2020 (5). We first filter these MPAs to those that are listed as fully “no-take,” and also those that are either “Designated” or “Established.” Implementation dates in MPA Atlas frequently do not correspond to the date when fishing bans begin, so for each MPA we also performed an extensive gray literature review using Google and Google Scholar to determine the exact date when the no-take zone was fully implemented and enforced. When the exact date could not be found, we next tried to determine the month, and if that could not be found the year. We used the search terms: “**mpa_name** no take implementation date” and “**mpa_name** implementation date.” Priority of dates was given to government management plans, followed by nongovernment organization reports, news articles, and blogs. This left us with 845 “no-take” MPAs in the analysis for which we had an MPA implementation date. To remain consistent with the MPA classification language provided in the MPA Guide (4), we refer to these MPAs in the text as “fully protected.”
  We then calculate 11 MPA-based model features, which vary spatially and temporally. First, we calculate the distance to the nearest MPA (=0 if the cell contains an MPA) and the years since that MPA was designated (=0 if the MPA was designated in the current year). The distance is calculated based on the distance to the nearest 1x1 degree rasterized version of the MPA boundary shapefile, while preventing travel through land masses. We recalculate distances for every pixel in every year because MPAs are implemented over time, so the nearest MPA will change from year to year. We expect that MPAs will have a larger effect on nearby pixels and that the effect may change over time following implementation (e.g., due to changes in enforcement, compliance, and/or transitions to new fishing grounds over time).
  Next, we quantify the fraction of MPA coverage for each pixel, its first-degree neighbor pixels, and its second-degree neighbor pixels. We also generate a categorical variable for whether a pixel is fully inside (pixel coverage = 100%), fully outside (pixel coverage = 0%), or partially covered by an MPA (pixel coverage >0% and <100%). Since fishing effort is allowed near the edges of MPAs, a pixel with only partial MPA coverage could have a smaller reduction in fishing effort than an MPA with full coverage; indeed, if there is significant “fishing the line” (aggregation of fishing effort near MPA boundaries to capture spillover), pixels with partial MPA coverage or near MPAs with any coverage could experience an increase, rather than a decrease, in fishing effort postimplementation.
  Then, we quantify the fraction of the current year that the MPA was in place. For pixels inside or partially covered by MPAs, the fraction =1 for full year coverage and is >0 but <1 for partial year coverage; for pixels outside MPAs, the fraction always =0. We expect that MPAs implemented later in the year will be associated with a smaller reduction in annual fishing effort. In fact, an MPA implemented later in the year could be associated with an increase in fishing effort if the announcement of a new MPA causes an anticipatory increase in fishing effort prior to implementation, an effect known as the “Blue Paradox” (17). To further capture anticipatory effects, we also include two Boolean features for whether an MPA will be implemented in a pixel in the following year or in two years, interacted with two Boolean features for whether the MPA will be fully or partially covered by that MPA (this procedure generates four unique features).
  In the special case where a pixel overlaps with multiple MPAs, the fraction of MPA overlap is based on the union of these MPAs, and the years since designation is based on the oldest MPA.
3. Environmental. 12 features capture environmental conditions that may influence fishing desirability. Sea surface temperature and sea surface temperature anomaly (46), chlorophyll-A (47), and wind speed (48) vary spatially and temporally, whereas indices for both the El Niño-Southern Oscillation (49) and Pacific Decadal Oscillation (50) vary only temporally. Since these variables all have higher spatial and/or temporal resolution than our final dataset, we calculate both the mean and SD across source pixels and months for each of our 1x1 degree pixel-years.
4. Geographic. Seven features capture geographic features of each pixel, which vary spatially but not temporally. We include numeric features for latitude, longitude, distance to shore, distance to the nearest seamount, and bathymetry depth. Global Fishing Watch provides distance to shore and bathymetry depth for the centroids of 0.01x0.01 pixels (24); we calculate the average of these values for our 1x1 degree pixels. Distance to the nearest seamount is based on the distance between each 1x1 degree pixel centroid and over 37,000 seamount point locations (51). Fish tend to aggregate near seamounts, so we expect pixels that are closer to seamounts to have more fishing effort. The relationship between fishing effort and distance to shore or bathymetry depth likely depends on gear type; trawlers tend to prefer closer, shallower waters, while longliners tend to prefer farther, pelagic waters. Latitude and longitude flexibly capture other elements of geographic location that we do not directly measure.
  We also include categorical variables for the ocean (52) and mesopelagic zone (53) of the pixel. If a pixel falls into more than one ocean or mesopelagic zone, the pixel is assigned to the category that covers the largest fraction of the pixel. These variables capture potential geographic differences in where commercially valuable fish tend to aggregate, which therefore influences fishing effort.
5. Governance. Six features describe the governance characteristics of a pixel, which vary spatially but not temporally. The first set of features concern the presence of EEZs, which may differ in their capacity to effectively manage fisheries. First, we intersect pixels with EEZs (54) and assign pixels to the EEZ with the largest pixel overlap using its sovereign ISO3 EEZ label. To reduce the dimensionality of the data, we group all EEZs that individually represent less than 1% of the pixels in the training data into an “other” category. Pixels that do not overlap with any EEZs are assigned an EEZ value of “high_seas.” Next, for each pixel, we calculate the fraction of the pixel area overlapping with its assigned EEZ (this takes a value of 0 for pixels in the high seas; for pixels that overlap multiple EEZs, this value is calculated using only the single EEZ with which it has the largest overlap). Then, we calculate the distance to the nearest 1x1 degree rasterized pixel of the EEZ boundary shapefile, while preventing travel through land masses (=0 for pixels fully inside EEZs). We also determine the name of this nearest EEZ sovereign state. We assign each pixel to one of 7 World Bank Development Indicators regions based on its EEZ (55); if there is no EEZ, it is assigned to the high seas. Then using the Global Fishing Index (GFI) (56), we assign each pixel to one of 11 governance capacity score categories based on its EEZ (the GFI does not have data for high seas areas, so for pixels in the high seas we assign a category value of “high_seas;” the GFI also does not have complete global coverage for all EEZs, so for pixels in EEZs that don’t have data from GFI we assign a category value of “no_data”).
6. Economic. Three features capture economic conditions that may affect the profitability of fishing, and therefore fishing effort. Distance to port, which varies spatially but not temporally, is a proxy variable for the cost of fishing, with farther trips tending to cost more. GFW provides distance to port for the centroids of 0.01x0.01 pixels (24); we calculate the average of these values for our 1x1 degree pixels. Fuel prices, which varies temporally but not spatially, also affect fishing costs. We calculate the mean and SD across months in the year of the Intermediate Fuel Oil (IFO) 380 fuel price (57). For each cost proxy, we expect that higher costs tend to reduce fishing effort.
7. Technological. Our fishing effort outcome variable is derived from satellite-based AIS vessel messages. Satellite-based AIS reception varies globally, and there are some regions with consistently poor reception (e.g., Southeast Asia). We capture the quality of AIS reception with two features that vary spatially: messages per day for Type A transponders and for Type B transponders (24). Type A transponders are higher quality and usually used by commercial vessels, whereas Type B transponders tend to be lower quality and used by recreational vessels.
8. Residual effects. Finally, we cannot capture every feature that determines the location and intensity of fishing effort in a pixel-year, so we include two features that capture a wide range of the residual (or otherwise unmeasured) effects. First, we calculate lagged AIS fishing effort [measured as log(h/m^2^), like our outcome variable], which varies spatially and temporally (24). We use a 1-, 2-, or 3-y lag, depending on the model’s forecast horizon (see details in the next section). We also include a numeric variable for the current year to flexibly capture time trends not otherwise defined by the temporally varying features.

### Model Training, Out-of-Sample Performance Testing, and Robustness Checks.

We train and test three separate models for three separate forecast horizons *t* (1, 2, and 3 y). For each forecast horizon and pixel-year observation, we predict future fishing effort in *t* years. To evaluate the performance of the three models, we separately perform both temporal and spatiotemporal out-of-sample testing using holdout testing datasets. The following steps apply to each of the three models.

1. For each pixel-year, modify the full dataset to create a new outcome variable for fishing effort in *t* years using time leads by pixel. Naturally, this reduces the size of the dataset—for any given year, there are only so many years in the future with existing data. Larger forecast horizons will therefore have smaller datasets to work with. For example, for *t*= 1, we use a dataset that includes rows of model features from 2020 and fishing effort in 2021, rows of model features from 2019 and fishing effort in 2020, etc. For *t*= 3, we use a dataset that includes rows of model features from 2018 and fishing effort in 2021, rows of model features from 2017 and fishing effort in 2020, etc.
2. For each of these three datasets, temporally split the data into a training dataset and a testing dataset. The testing dataset contains all data in the last year of the data, while the training dataset contains all data from the previous years. We can therefore test the temporal out-of-sample performance of the model to assess how well it predicts future years that were unseen during the model training. This helps us understand how well the model will perform in our simulations, which predict fishing effort in future years that have not yet been observed. *SI Appendix*, Fig. S1 shows the years represented in the training and testing datasets for each ocean and forecast horizon. *SI Appendix*, Fig. S2*A* shows the resulting dataset sizes, by region (inside MPA, partial MPA overlap, or outside MPAs). *SI Appendix*, Fig. S2*B* shows the number of distinct MPAs represented in each of these datasets, by region (i.e., distinct MPAs that are fully covered by inside MPA pixels, MPAs that are partially covered, and MPAs that are the nearest MPA to outside MPA pixels).
3. Using the training dataset, create CV partitions which each have an analysis (i.e., training) split and an assessment (i.e., testing) split (*SI Appendix*, Fig. S3). We use these for optimizing hyperparameters for a Stage 1 model (classification, or extensive margin), and then for optimizing hyperparameters for a Stage 2 model (regression, or intensive margin). We create a series of time-based folds where the assessment split comes from the last year of data and the analysis split uses the preceding year of data. In this way, the folds allow us to independently test the CV performance in predicting different years from the training dataset. We use the *timetk* R package to implement time-based CV splitting (58).
4. For each of the CV partitions, train and test random forest models (59) across a grid of 10 hyperparameter combinations. We test an entropy-maximizing grid over two hyperparameters—*mtry* (the number of features that will be randomly sampled at each tree split node) and *min_n* (the minimum number of observations required for further splitting each node). We use 500 trees for all random forest models. We independently optimize hyperparameters separately for both the Stage 1 and Stage 2 models, so that each gets its own set of hyperparameters. Note that the Stage 1 model uses all observations from the analysis partition for training, while the Stage 2 model is conditional on there being fishing effort and therefore uses only those observations with a nonzero fishing effort from the analysis partition for training. We use the *ranger* R package to implement random forests (60).
5. For the Stage 1 model, we choose the optimized hyperparameter set that maximizes ROC area-under-the curve (*roc_auc*), a model performance metric that measures the model’s general ability to differentiate between two classes and is agnostic to the classification cutoff threshold. We calculate disaggregated *roc_auc* separately for each CV fold and then average it across folds.
6. For the Stage 1 model and using its optimized hyperparameter set, we further choose the optimal classification cutoff threshold that maximizes the F1 score (*f_meas*), which is the harmonic mean of *precision* and *recall*. Precision and recall depend on the number of true positives (TP) and false negatives (FN). Precision is TP/(TP + FP), and recall is TP/(TP + FN).
7. For the Stage 2 model, we choose the optimized hyperparameter set that maximizes *rsq_trad*. *rsq_trad* is calculated using the traditional definition of R squared which uses sum of squares, and allows for negative values. This is opposed to *rsq* which forces the values to be between 0 and 1. *rsq_trad* is therefore a more conservative estimate of model performance (61). We calculate the backtransformation smearing coefficient for each fold based on the predictions from the training split, and then backtransform the test split predictions to get level predictions. Using these level predictions, we calculate *rsq_trad* for each CV fold and then summarize it as the average *rsq_trad* across folds.
8. Using the optimized hyperparameter sets for the Stage 1 and Stage 2 models, we train models using the full training dataset. For the Stage 2 models, we calculate the backtransformation smearing coefficient using the training data and then backtransform the testing data predictions using this value to get level predictions.
9. Using the Stage 1 and Stage 2 trained models, we make out-of-sample predictions for the testing dataset, which has not been used up to this point. For Stage 1 predictions, we use the optimized classification threshold cutoff for classifying each prediction.
10. We combine each Stage 1 and Stage 2 out-of-sample predictions into full hurdle model predictions. For each observation, we simply multiply the stage 1 classification prediction (0 or 1) by the stage 2 prediction (h/m^2^ of fishing effort).
11. For the Stage 1 model, we calculate out-of-sample *roc_auc*, *f_meas*, *precision*, and *recall*. We do this for all pixels globally, as well as 3 mutually exclusive regions (inside MPAs, partial overlap, and outside MPAs). This gives us the Stage 1 performance for predicting out-of-sample future fishing effort (*SI Appendix*, Fig. S4*A* and Table S1).
12. To quantify stage 2 performance for predicting future fishing effort, we calculate out-of-sample *rsq_trad*, *rsq*, *rmse* (root-mean-square error), and *nrmse* (normalized root-mean-square-error, which is *rmse* divided by the SD of the outcome in the testing data). Note that *rsq_trad*, *rsq*, and *nrmse* are unitless, while *rmse* is in units of the outcome variable (h/m^2^). We do this for all pixels globally to obtain global performance metrics, as well as for three mutually exclusive regions (inside MPAs, partial MPA overlap, and outside MPAs) (*SI Appendix*, Fig. S4*B* and Table S1).
13. We train the final Stage 1 and Stage 2 models using the entire dataset (which uses data from all years). Again, we use CV to tune hyperparameters for each model, as in Steps 3 to 8. For the Stage 2 model, we calculate the final backtransformation smearing coefficient using the entire dataset. These final Stage 1 and Stage 2 models, in combination with the final backtransformation smearing coefficient, will be used to make predictions in the simulations.
14. As an additional out-of-sample performance test, we repeat Steps 2 to 12, but this time doing a spatiotemporal training/testing data split instead of just a temporal split. For this test, we split the data into 10 different training datasets and 10 matched leave-one-location-out testing datasets, one for each ocean. The testing dataset for each ocean contains all data in the last year of the data, while the training dataset contains all data from the other nine oceans in the previous years. In this way, we test the spatiotemporal out-of-sample performance of the model to assess how well it predicts future years in spatial areas that were unseen during the model training. This helps us understand how well the model will perform in our simulations, which predict fishing effort in future years that have not yet been observed, and in areas that have new MPAs but which did not have MPAs in the training dataset. Using these 10 splits, we build and test 10 optimized models using the full training and tuning procedure outlined in Steps 3 to 12. This gives us out-of-sample performance measures for each of our 10 models corresponding to the out-of-sample performance in each of the 10 holdout testing oceans (*SI Appendix*, Fig. S5).
15. To test the robustness of the baseline model specification from Steps 1 to 12 (random forest with all model features), we test three additional model specifications: 1) a random forest with just the model feature of lagged log fishing effort (using the entire procedure described above); 2) logistic regression for stage 1 (using the *stats::glm* function) and linear regression for stage 2 (using the *stats::lm* function) (62), with all model features (similar to what is described above, but without cross-validation since these models have no hyperparameters to tune); and 3) logistic regression for stage 1 and linear regression for stage 2, with just the model feature of lagged log fishing effort (again similar to what is described above, but without cross-validation since these models have no hyperparameters to tune) (*SI Appendix*, Fig. S6).

### Model Interpretability.

To better understand why the model gives certain predictions and to increase the model’s interpretability, we calculate Shapley values for the final trained Stage 1 and Stage 2 models (i.e., the models that are trained using the entire dataset). Shapley values quantify the absolute contribution of each feature toward the prediction of each individual observation (i.e., it is a measure of local explanation) (63). To translate local observation-level explanations into measures of global explanation, we:

1. We select a random sample of 1,000 observations from the training dataset, and a random sample of 100 observations to represent the background data. For each observation, we use the Kernel SHAP method to calculate the approximate Shapley value, using the *kernelshap* R package (64). For each feature, we take the mean absolute value from across the observations to obtain a single Shapley value (*SI Appendix*, Fig. S7).
2. We then group the features into the seven general categories defined in *Data processing and feature engineering* and Table 1 (except including the previous fishing effort and year features individually); based on the additive property of Shapley values, we sum the observation-wise values across features for each feature group and observation, and then take the mean absolute feature group values from across all observations to obtain a single Shapley value per feature group (*SI Appendix*, Fig. S8). We use the following groups: Previous fishing effort; Geographic (spatial geographic features including bathymetry depth, distance to shore, mesopelagic region, ocean, latitude, and longitude); Environmental (spatiotemporal and temporal environmental features, including sea surface temperature (SST), SST anomaly, wind speed, chlorophyll concentration, El Niño–Southern Oscillation (ENSO) and Pacific Decadal Oscillation (PDO) indices); Technological (class A and class B AIS reception); MPA Implementation (MPA-related features); Governance (EEZ indicators and Global Fishing Index governance capacity index); Economic (distance to port and fuel price); and Year.
3. We create dependence plots for each feature that plot the Shapley value against the feature value (*SI Appendix*, Figs. S9 and S10).

### Simulations.

1. Using the final trained Stage 1 and Stage 2 models and smearing coefficient, make predictions for each of the three forecast horizons using the actual fully protected MPA network observed in 2020 (which covers 2.5% of the oceans). This is our BAU scenario of what would happen in a world without any new MPAs. Since the predicted outcome variable is area-normalized effort in h/m^2^, we multiply this number by the area of each pixel to get predicted fishing hours per pixel.
2. Using the final trained Stage 1 and Stage 2 models and smearing coefficient, make predictions for each of the three forecast horizons under a series of hypothetical MPA networks. These networks were designed for different objectives, and each covers different percentages of the world’s oceans, from current levels up to 30%. For each of these hypothetical networks, we include the locations of current fully protected MPAs from 2020 in order to be able to directly compare predictions to the BAU counterfactual scenario (which only includes fully protected MPAs from 2020):
  – Networks from Sala et al. 2021 (20): We consider four network types from this analysis that each prioritize different objectives: biodiversity protection; carbon sequestration; food provision; and a multiobjective network that equally weights all three objectives. For each network type, we use seven different protection targets: 3%, 5%, 10%, 16%, 20%, and 30%.
  – Network to protect EBSAs (27, 65): This network was proposed by an expert-driven process facilitated by the Convention on Biological Diversity, and covers roughly 20% of the world’s oceans.
  – Network in Visalli et al. 2020 (26): This network proposes areas for priority protection in marine areas beyond national jurisdiction and covers roughly 16% of the world’s oceans.
  – Random: We randomly rank the pixels and successively close pixels until a desired target is reached. We use 3%, 5%, 10%, 16%, 20%, and 30% area protected targets.
  – Most-fished areas: We first rank pixels in descending order by the amount of fishing effort (hours) in 2020. We use 3%, 5%, 10%, 16%, 20%, and 30% area protected targets. For each target, we close the top most-fished pixels necessary to achieve the target.
  – Unfished areas: We first select pixels that have zero fishing effort in 2020. We use 3%, 5%, 10%, 16%, 20%, and 30% area protected targets. For each target, we close a random sample of these unfished pixels necessary to achieve the target.
3. For each hypothetical MPA network and forecast horizon, calculate the absolute and percentage difference between the hypothetical MPA network predictions and the BAU network predictions. We calculate these differences for all pixels globally, as well as for the three mutually exclusive pixel regions (fully inside MPAs, partial MPA overlap, and fully outside MPAs).

## Supplementary Material

Appendix 01 (PDF)

## Data Availability

All code necessary for reproducing the analysis can be found at https://zenodo.org/records/11625791 (66). Most data necessary for reproducing the analysis are also available directly in this repository, including our fishing effort outcome variable and all model feature data except those relating to bunker fuel prices. The bunker fuel price data used in our analysis are subject to restricted use and are not available for public redistribution. Information on obtaining these data directly from Bunker Index can be found at https://bunkerindex.com/. We use the R programming language version 4.3.3 for all code (62). We use the *targets* package to ensure full reproducibility of the data processing and analysis pipeline (67), and the *renv* package to ensure a reproducible package environment (68). We use the *tidyverse* suite of packages for all data wrangling tasks (69), and the *tidymodels* suite of packages for the general machine learning framework (70). We use functions from the *yardstick* package for calculating all model performance metrics. For spatial operations, we use the *sf* (71), *terra* (72), *stars* (73), *exactextractr* (74), and *raster* (75) packages. For all spatial joins, area calculations, and distance calculations, we use the Mollweide equal-area projection.
