# Supplementary material for: Global expansion of marine protected areas and the redistribution of fishing effort
Source: Proc Natl Acad Sci U S A. 2024 Jul 9;121(29):e2400592121. doi: 10.1073/pnas.2400592121 (PMC11260147; doi:10.1073/pnas.2400592121)
Supplement: Supplementary file 1 — Appendix 01 (PDF) [file pnas.2400592121.sapp.pdf]

Supporting Information for

Global expansion of marine protected areas and the redistribution of fishing effort

Gavin McDonald, Jennifer Bone, Christopher Costello, Gabriel Englander, Jennifer Raynor

Corresponding Author: Gavin McDonald
E-mail: gmcdonald@bren.ucsb.edu

This PDF file includes:

Supporting text
Figs. S1 to S15
Table S1
SI References

Supporting Information Text

Materials and Methods. The first set of figures and tables in the Supporting Information are those referenced in the Materials and Methods section of the main text (Figs. S1 - S10 and Table S1). The next figures show our simulation prediction results disaggregated by forecast horizon region (inside MPAs, partial overlap with MPAs, outside MPAs, and global), both in terms of the number of fished pixels (Fig. S11) and in terms of the amount of effort (Fig. S12).

Palau case study. We then use Palau as a case study to look at the historically observed fishing effort from 2016-2021 inside and outside the Palau National Marine Sanctuary, which was implemented in 2020 (Fig. S13). We do this for the two largest fishing fleets in Palau, Taiwan and Japan. We also consider the fleets of Taiwanese and Japanese vessels that fished in Palau prior to the MPA implementation (which we call the "pre-MPA fishing fleet") and look at where these vessels fished before and after the MPA implementation (Fig. S14).

Results by Large Marine Ecosystem. For each each Large Marine Ecosystem (1) and MPA network scenario of 30% coverage, we: 1) calculate the predicted total business-as-usual counterfactual fishing hours after a 3 year time horizon; 2) calculate the predicted total fishing hours in the MPA network scenario after a 3 year time horizon; and 3) calculate the percentage change in fishing hours in the MPA network scenario relative to the business-as-usual (Fig. S15).

Table S1. Temporal out-of-sample global performance for Stage 1 classification models and Stage 2 regression models, for each of the three forecast horizon models.

Model	Metric	Forecast horizon: 1	Forecast horizon: 2	Forecast horizon: 3
Stage 1	f_meas	0.912	0.909	0.91
Stage 1	precision	0.9	0.889	0.907
Stage 1	recall	0.924	0.931	0.913
Stage 1	roc_auc	0.97	0.967	0.969
Stage 2	nrmse	0.409	0.469	0.423
Stage 2	rmse	6.10e-07	6.98e-07	6.31e-07
Stage 2	rsq	0.898	0.853	0.861
Stage 2	rsq_trad	0.832	0.78	0.821

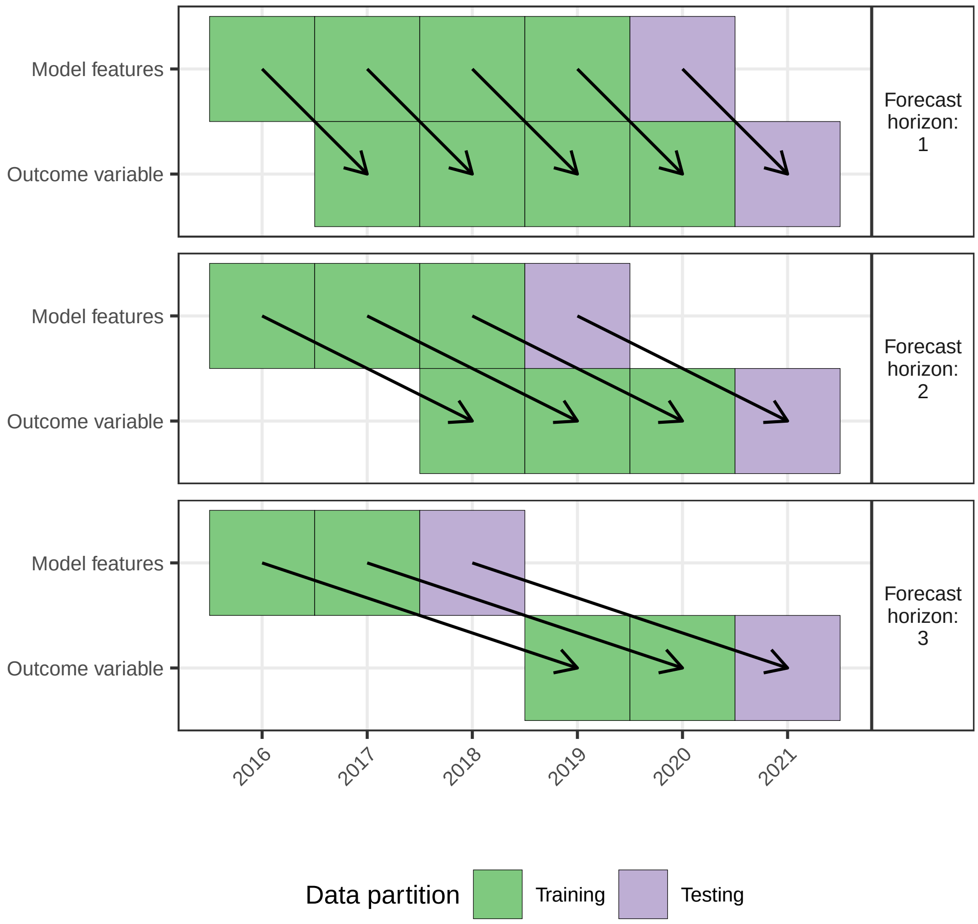

Fig. S1. Summary of the years contained in the training and testing datasets for each forecast horizon (this is the same for each of the ten out-of-sample oceans). The rows of each dataset contain numerous model features (e.g., sea surface temperature) and a single outcome variable (i.e., $hours/m^2$). The arrows indicate which outcome variable year is being predicted by which model feature year. For example, in the training dataset for a forecast horizon of 1, model features from 2016 are used to predict fishing effort in 2017; in the testing dataset for a forecast horizon of 2, model features from 2019 are used to predict fishing effort in 2021. All testing datasets are predicting fishing effort in 2021.

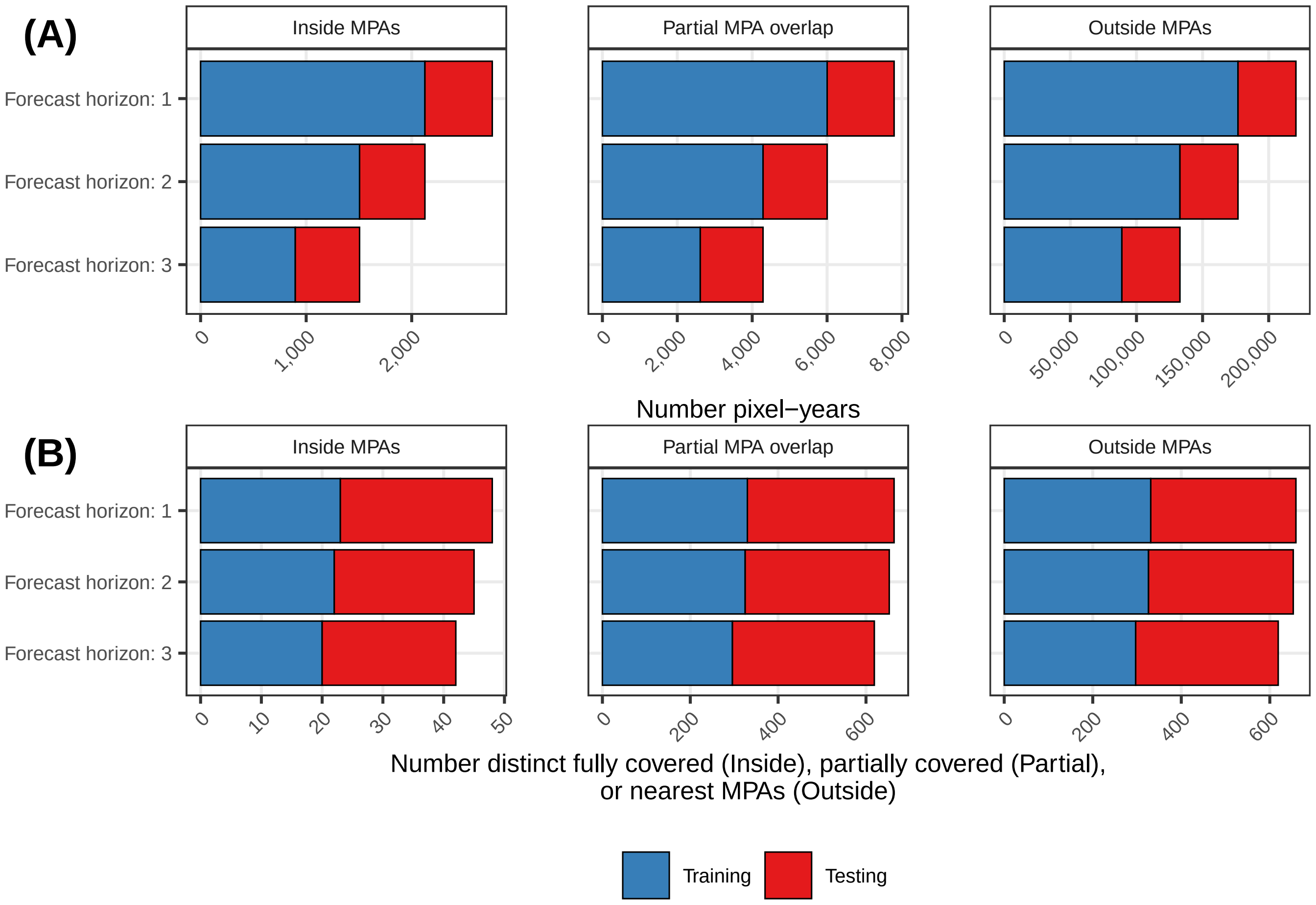

Fig. S2. Summary of the (A) training and temporal out-of-sample testing dataset observations (pixel-years), and (B) the number of distinct MPAs represented in the training and temporal out-of-sample testing datasets. Summaries are shown for each of the three forecast horizon models, by region (i.e., distinct MPAs that are fully covered by inside MPA pixels, distinct MPAs that are partially covered, and distinct MPAs that are the nearest MPA to outside MPA pixels).

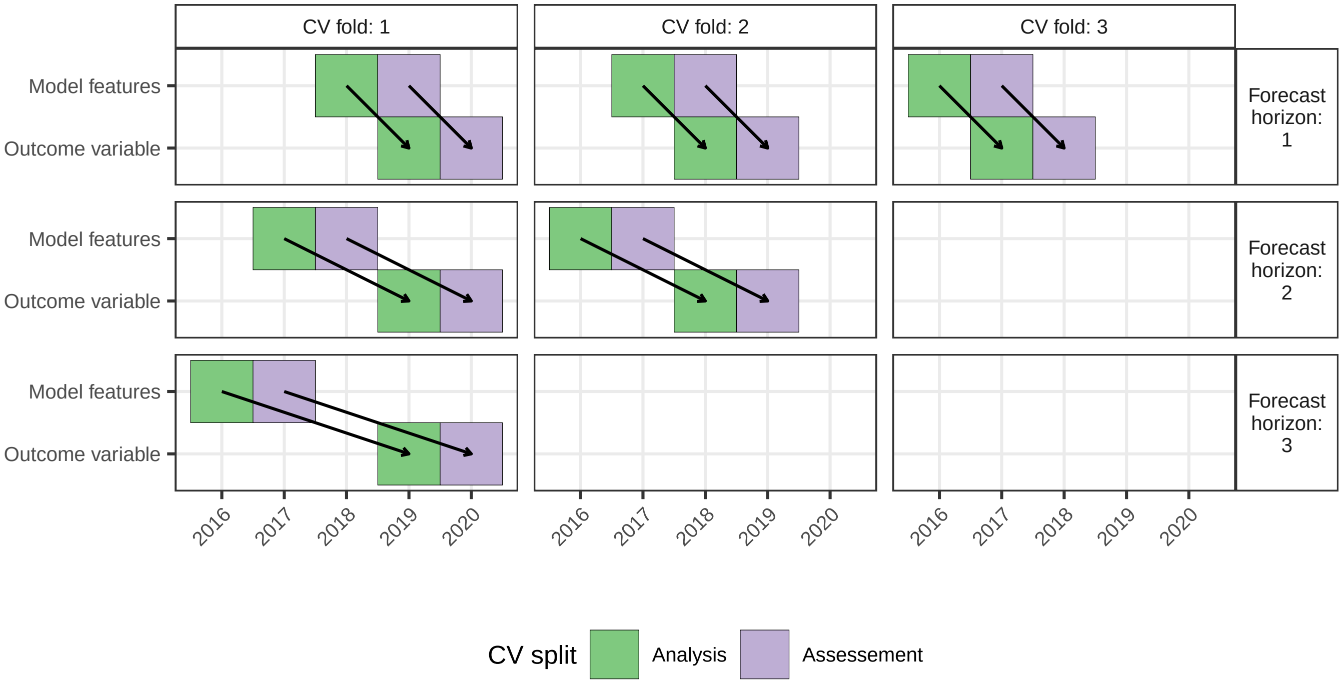

Fig. S3. Summary of the years represented in the cross-validation (CV) assessment and analysis splits for each fold and forecast horizon (this is the same for each of the ten out-of-sample oceans). The rows of each split contain numerous model features (e.g., sea surface temperature) and a single outcome variable (i.e., $hours/m^2$). The arrows indicate which outcome variable year is being predicted by which model feature year. For example, in the analysis split for CV fold 1 and a forecast horizon of 1, model features from 2019 are used to predict fishing effort in 2020; in the assessment split for CV fold 2 and forecast horizon of 2, model features from 2017 are used to predict fishing effort in 2019.

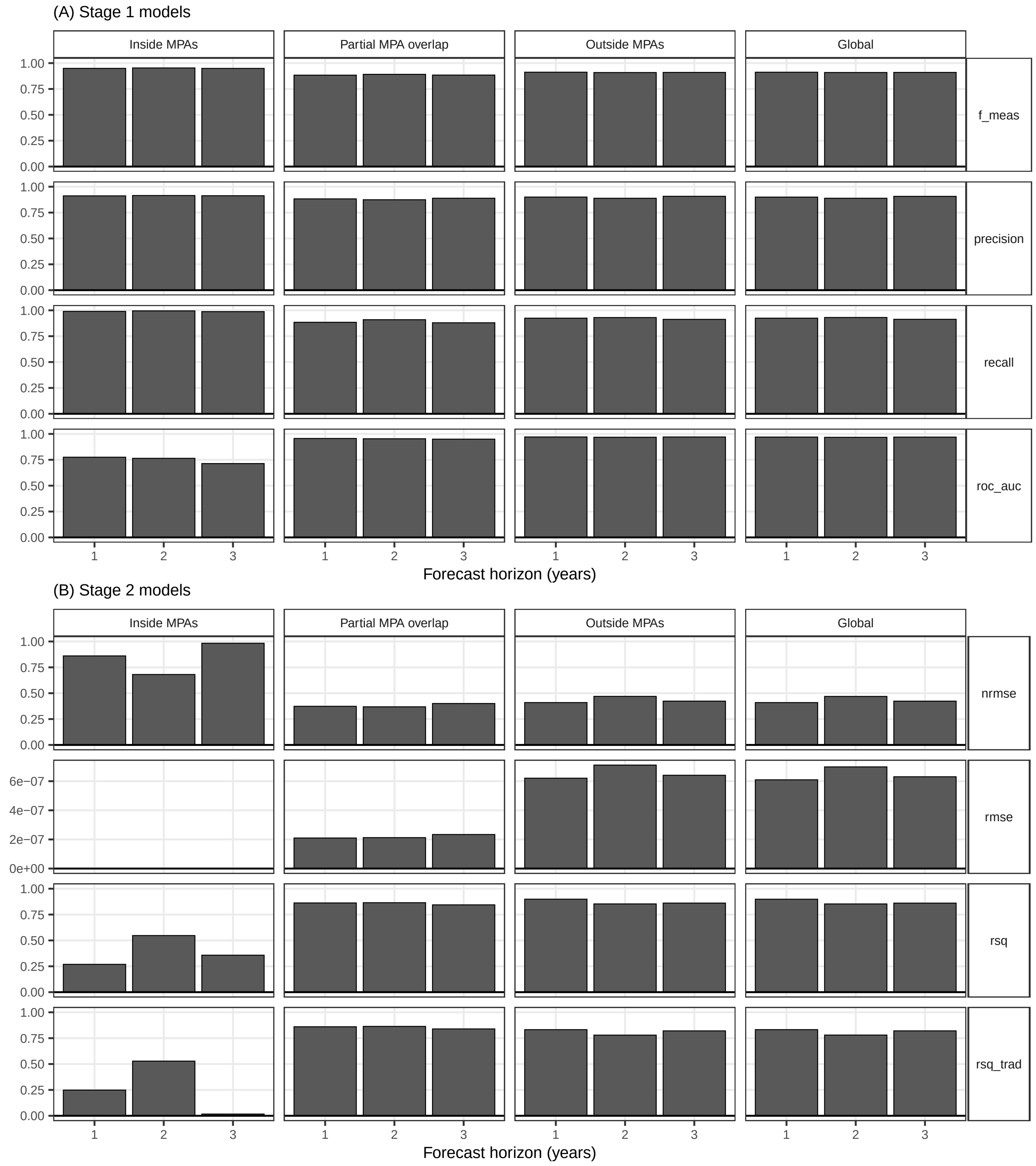

Fig. S4. Temporal out-of-sample performance for (A) Stage 1 classification models and (B) Stage 2 regression models, for different forecast horizons, performance metrics, and regions (the Global region aggregates all pixels).

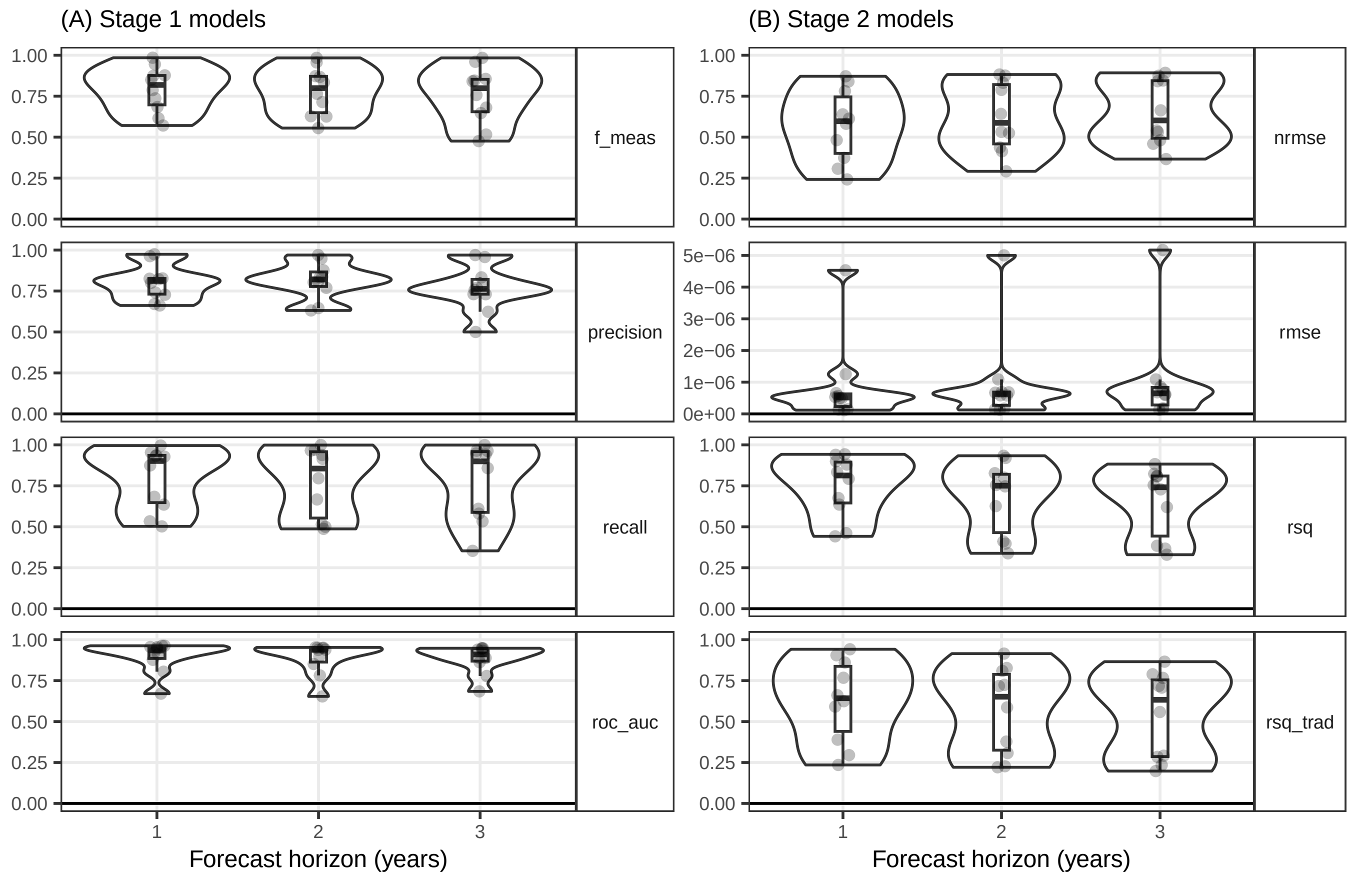

Fig. S5. Spatiotemporal out-of-sample performance for (A) Stage 1 classification models and (B) Stage 2 regression models for each forecast horizon and different performance metrics. Each point represents a different out-of-sample testing ocean, and the points are jittered to avoid visual overlap. Boxplots and violin plots show distributions of values from across the ten testing oceans. The boxplots for each bin show the median, 25th percentile, and 75th percentile.

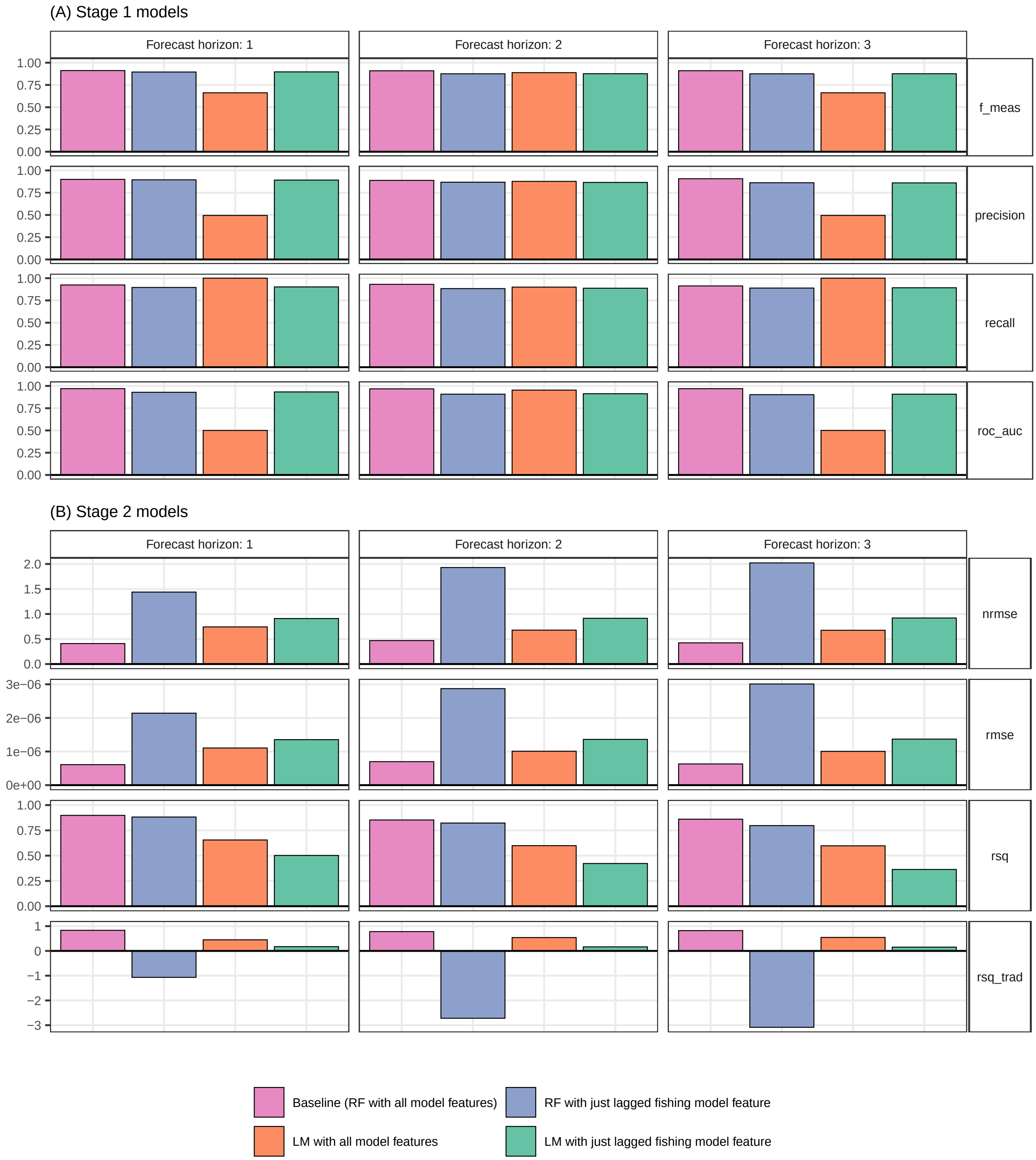

Fig. S6. Temporal out-of-sample performance for (A) Stage 1 classification models and (B) Stage 2 regression models, using four model specifications: 1) baseline using random forest (RF) with all model features; 2) random forest (RF) with just the model feature of lagged log fishing effort; 2) linear model (LM) using logistic regression for stage 1 and linear regression for stage 2, with all model features; and 4) linear model (LM) using logistic regression for stage 1 and linear regression for stage 2, with just the model feature of lagged log fishing effort.

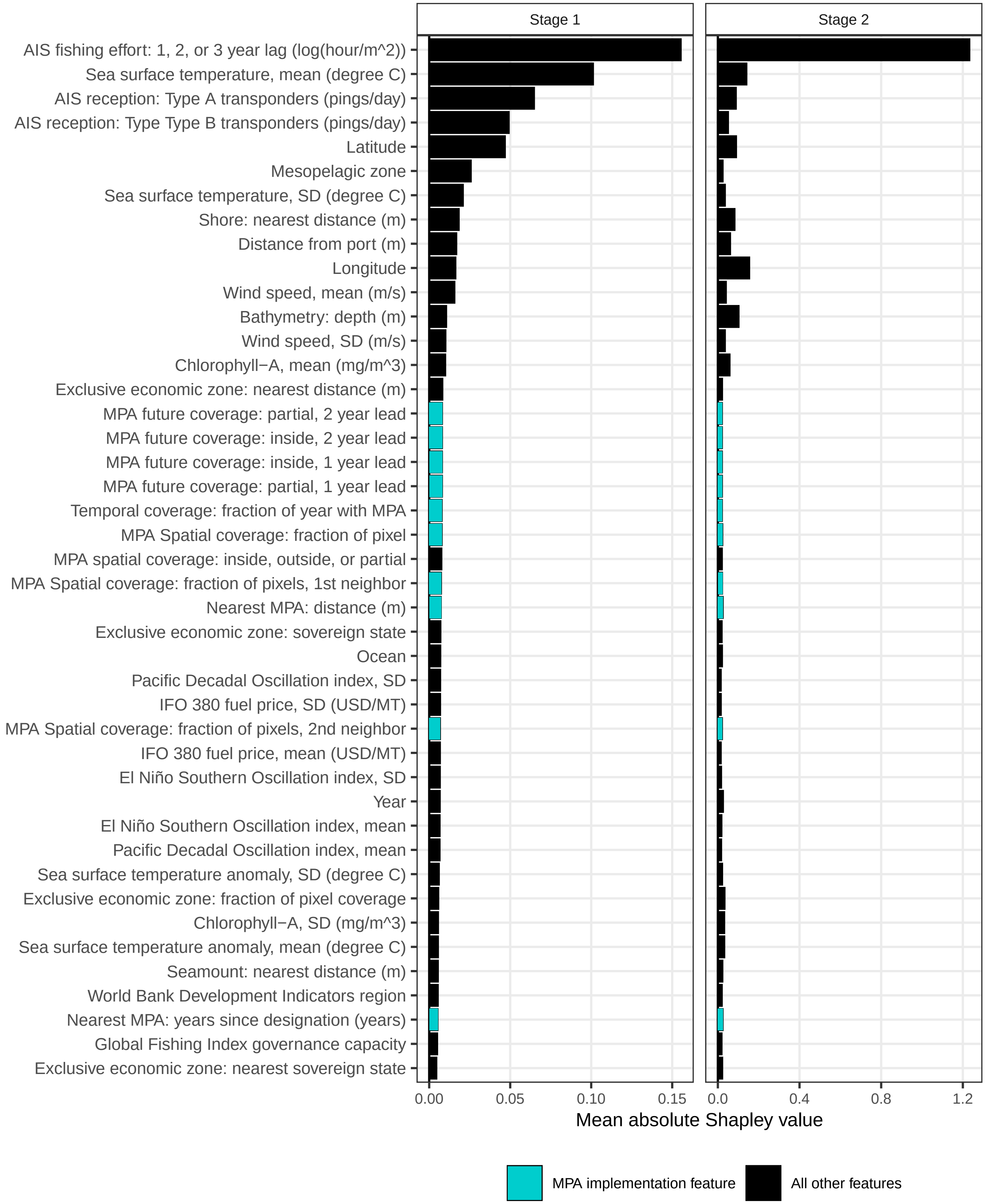

Fig. S7. Mean absolute Shapley values from final trained Stage 1 and Stage 2 models for a 1 year forecast horizon. Bars are color-coded by whether or not the feature is related to MPA implementation. Bars are arranged in descending order based on the feature importance from the Stage 1 model.

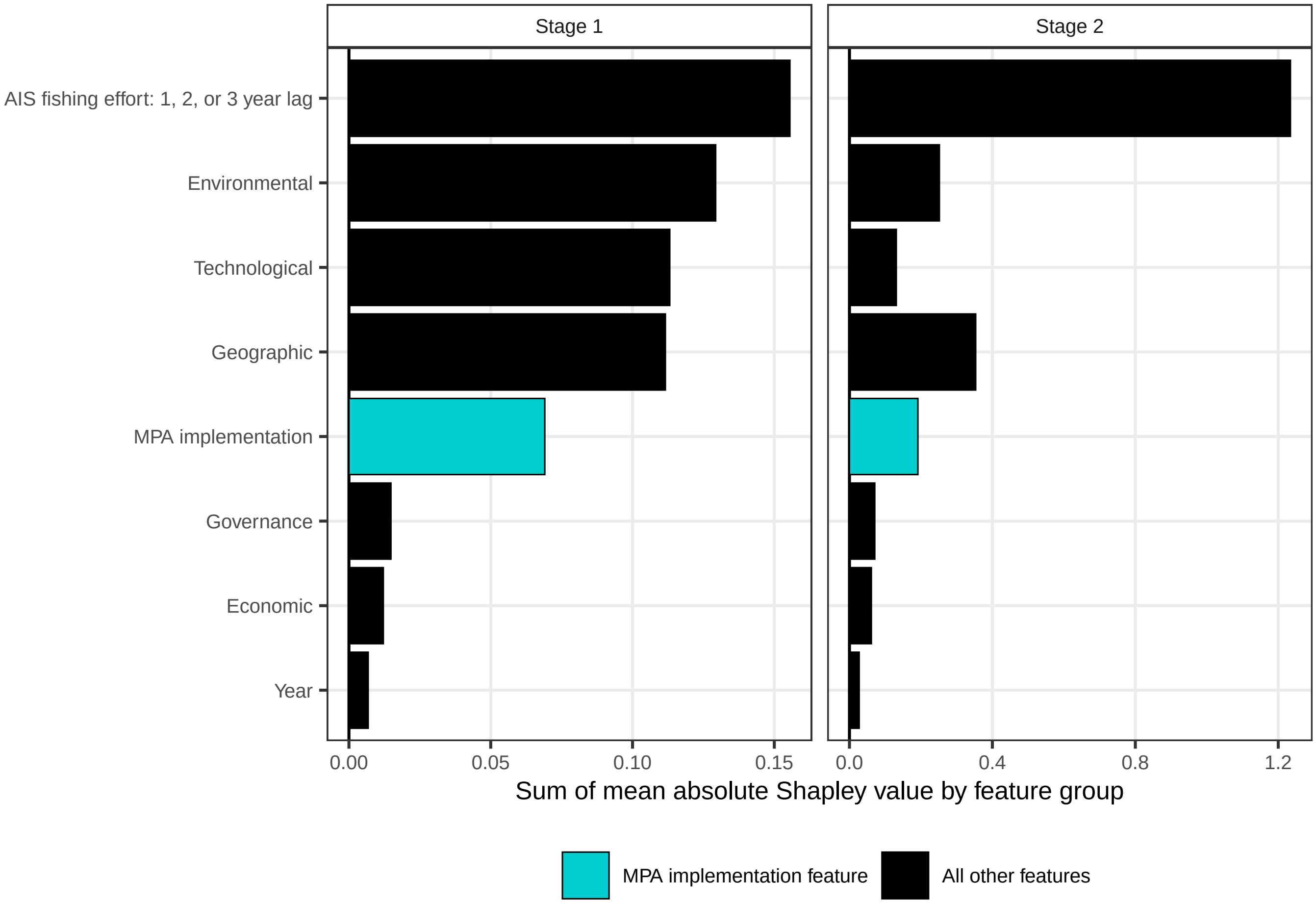

Fig. S8. Sum of mean absolute Shapley values by feature group from final trained Stage 1 and Stage 2 models for a 1 year forecast horizon, aggregated by feature group. We use the following groups: Previous fishing effort; Geographic (spatial geographic features including bathymetry depth, distance to shore, mesopelagic region, distance to seamount, ocean, latitude, and longitude); Environmental (spatiotemporal and temporal environmental features, including sea surface temperature (SST), SST anomaly, wind speed, chlorophyll concentration, ENSO and PDO indices); Technological (class A and class B AIS transponder, reception); MPA implementation (MPA-related features); Governance (EEZ sovereign state, nearest EEZ sovereign state, distance to nearest EEZ World Bank region, and Global Fishing Index governance capacity index); Economic (distance to port and fuel price); and Year. Bars are arranged in descending order based on the feature importance from the Stage 1 model.

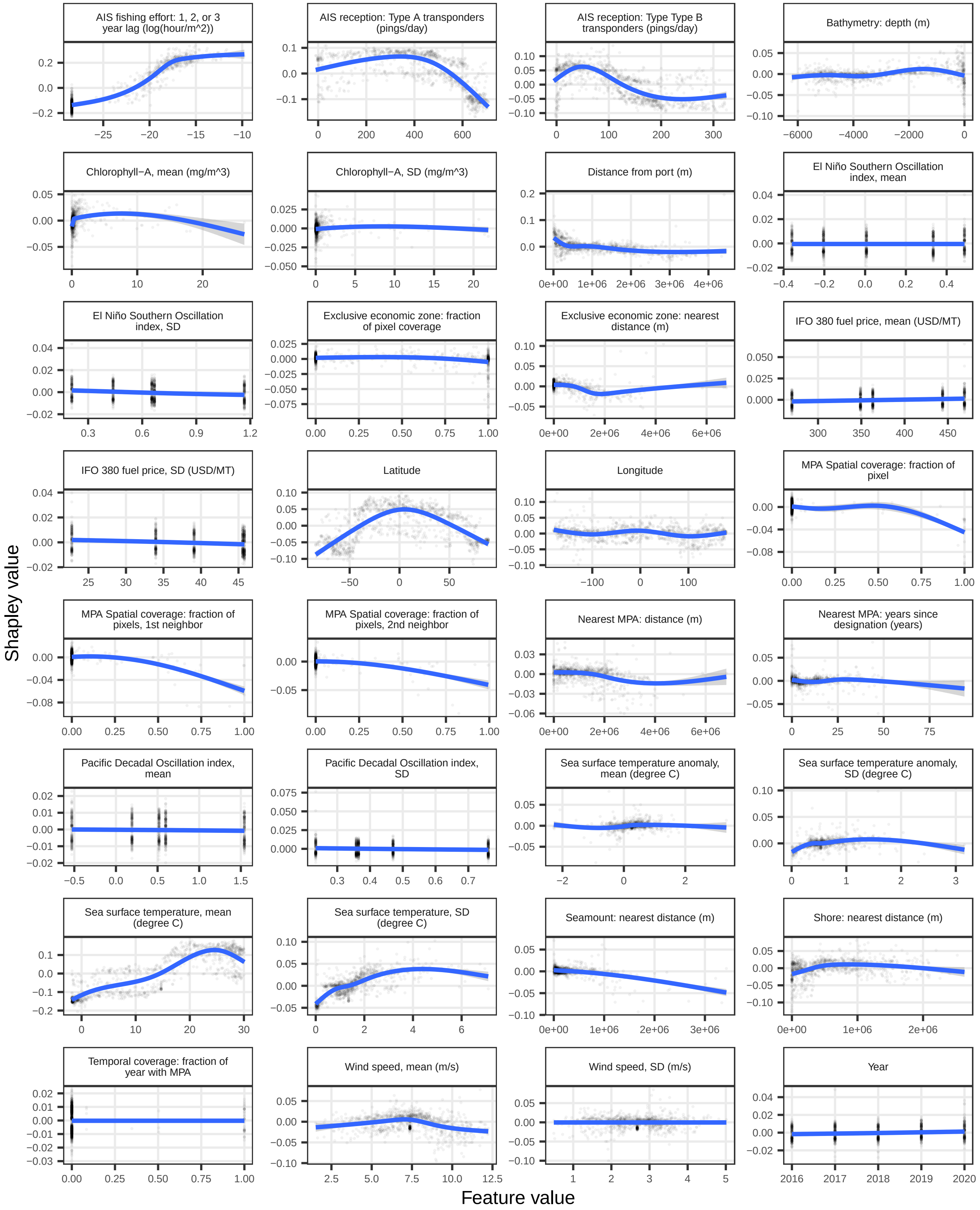

Fig. S9. Shapley value dependence plots from final trained Stage 1 model for all numeric model features and a 1 year forecast horizon. Each point represents the Shapley value from an individual observation, and the blue line uses a Generalized Additive Model smoothing function.

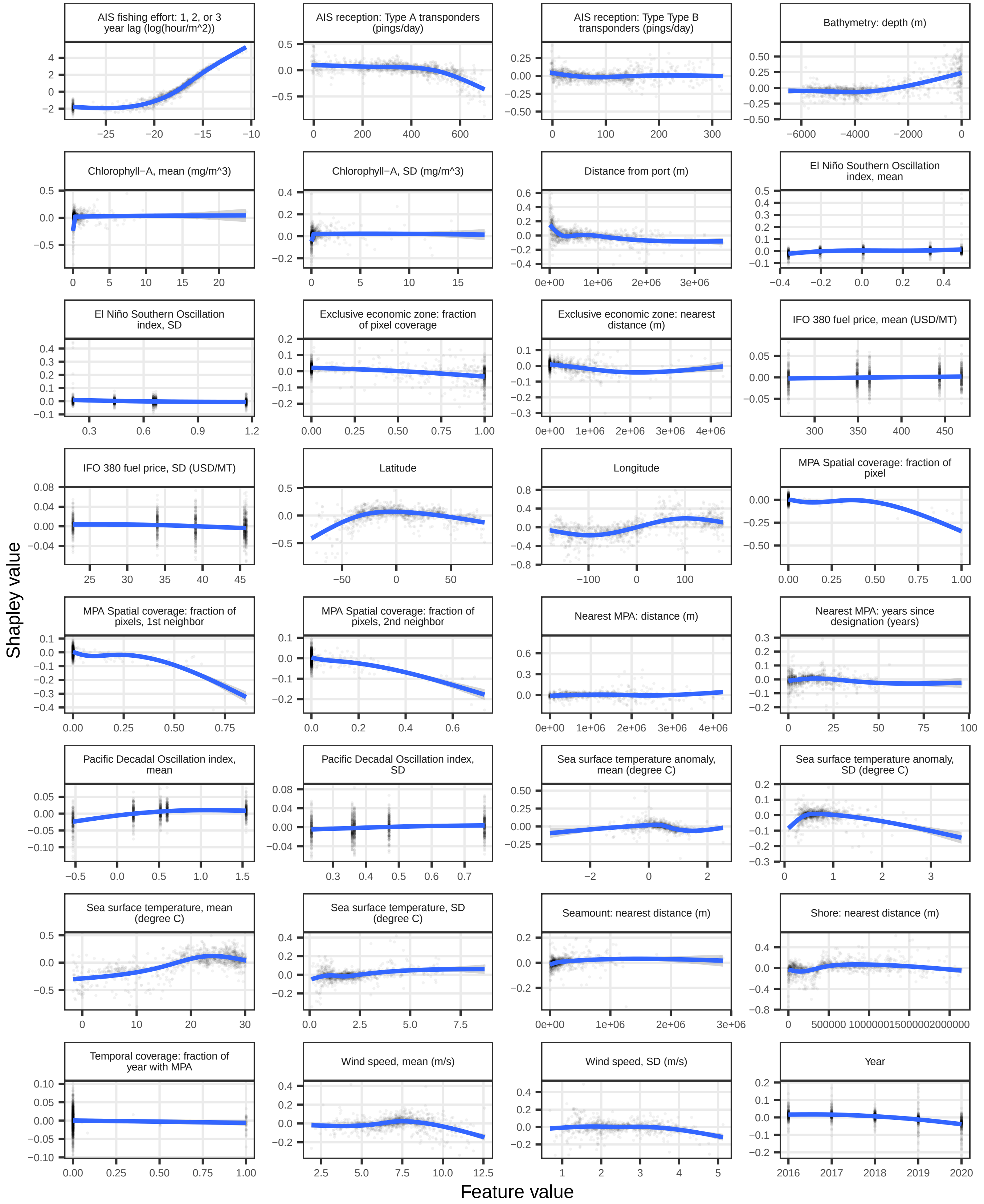

Fig. S10. Shapley value dependence plots from final trained Stage 2 model for all numeric model features and a 1 year forecast horizon. Each point represents the Shapley value from an individual observation, and the blue line uses a Generalized Additive Model smoothing function.

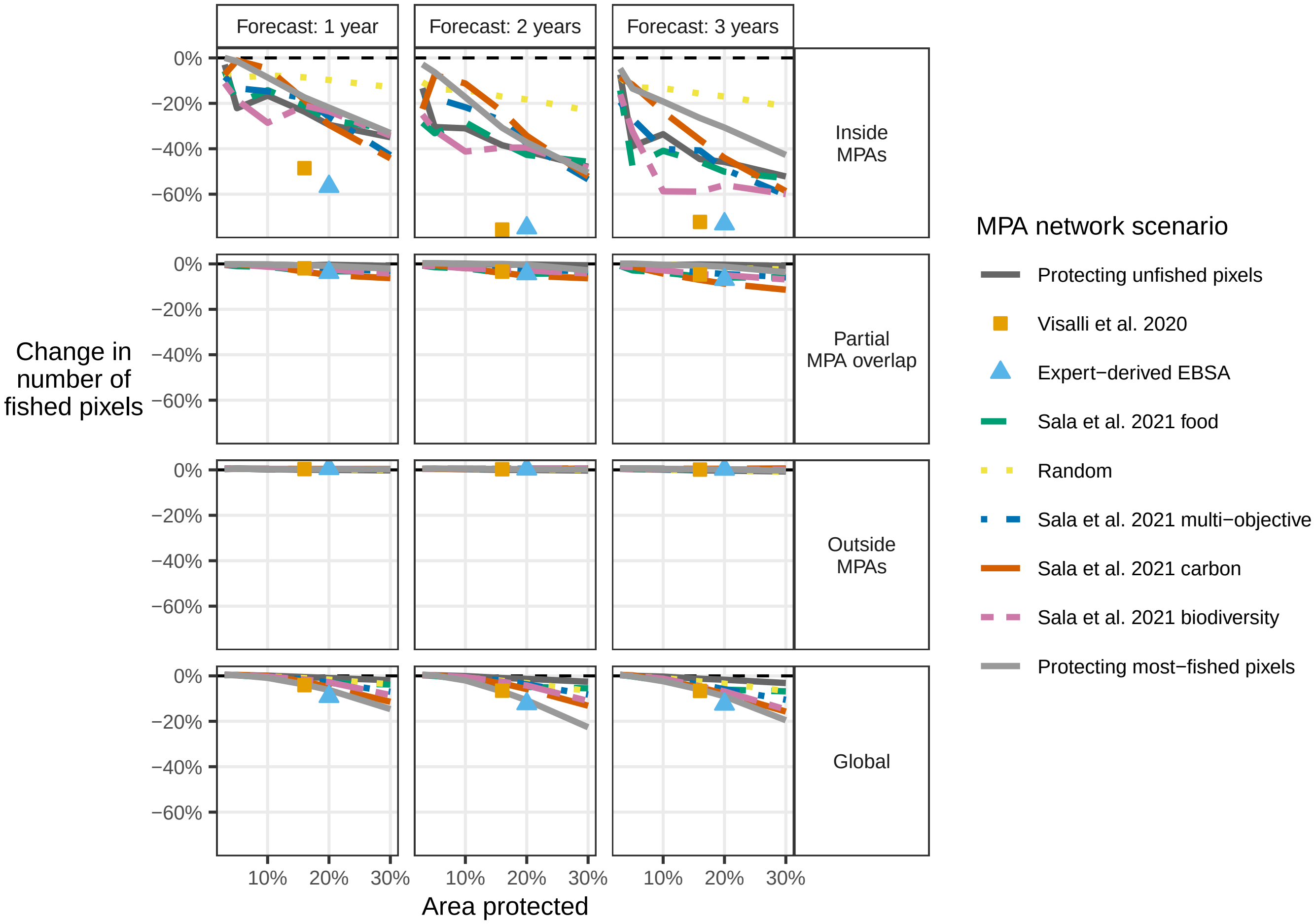

Fig. S11. Simulation results for the number of fished pixels (i.e., the Stage 1 model predictions). The y-axis shows the relative percentage difference between the MPA network scenario and the business-as-usual scenario. The x-axis shows the percent area of global oceans protected. The left-to-right panels represent the three model forecast horizons, and the top-to bottom panels represent results for pixels from the three mutually exclusive regions (inside MPAs, partial MPA overlap, and outside MPAs), as well as all pixels globally. Colors differentiate the various hypothetical MPA networks. Linetypes are used to further differentiate networks that can have various levels of protection, while shapes are used to differentiate networks that only have a single level of protection.

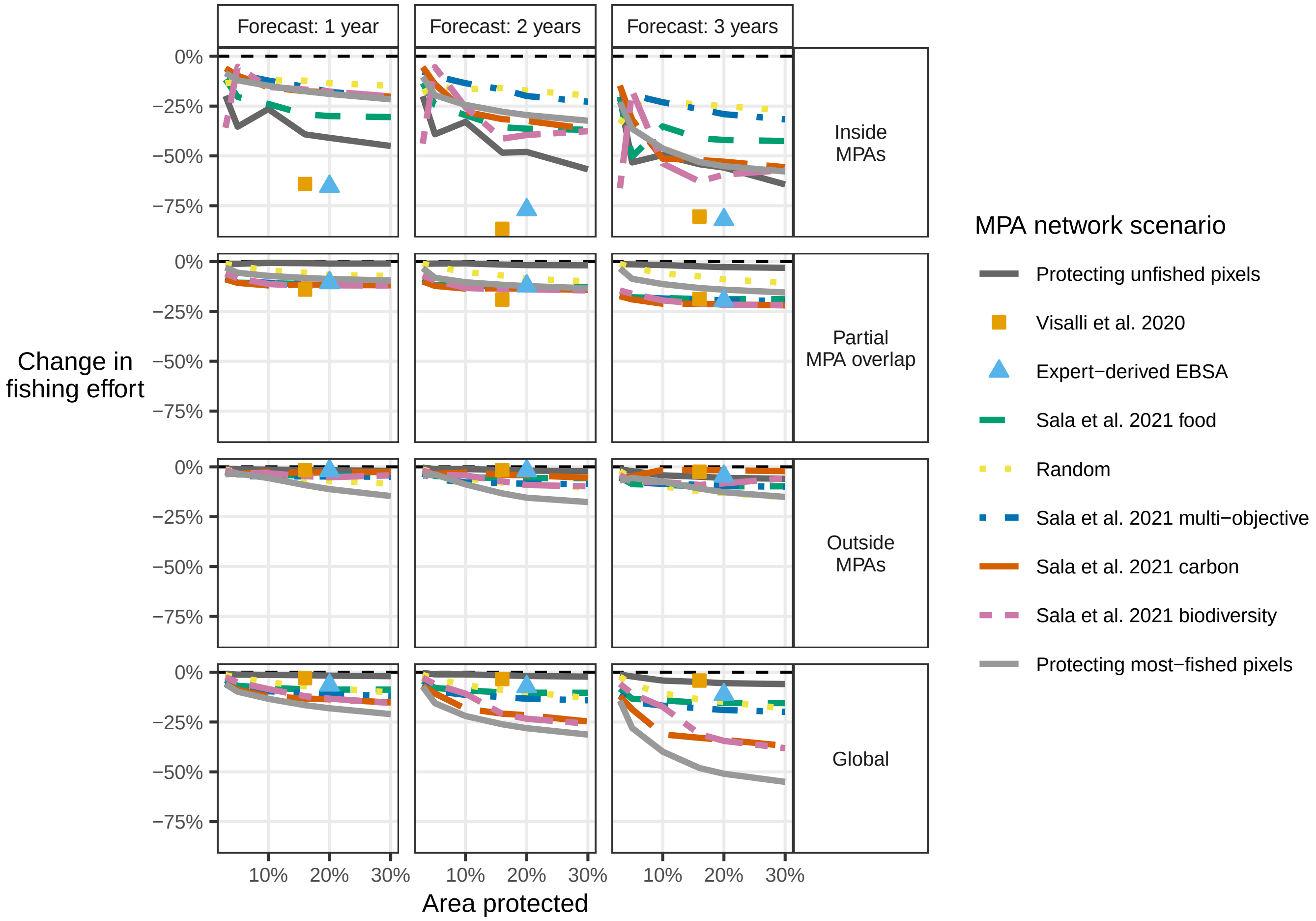

Fig. S12. Simulation results for the amount of predicted fishing effort for each MPA scenario from the full hurdle model. The y-axis shows the relative percentage difference between the MPA network scenario and the business-as-usual scenario. The x-axis shows the percent area of global oceans protected. The left-to-right panels represent the three model forecast horizons, and the top-to bottom panels represent results for pixels from the three mutually exclusive regions (inside MPAs, partial MPA overlap, and outside MPAs), as well as all pixels globally. Colors differentiate the various hypothetical MPA networks. Linetypes are used to further differentiate networks that can have various levels of protection, while shapes are used to differentiate networks that only have a single level of protection.

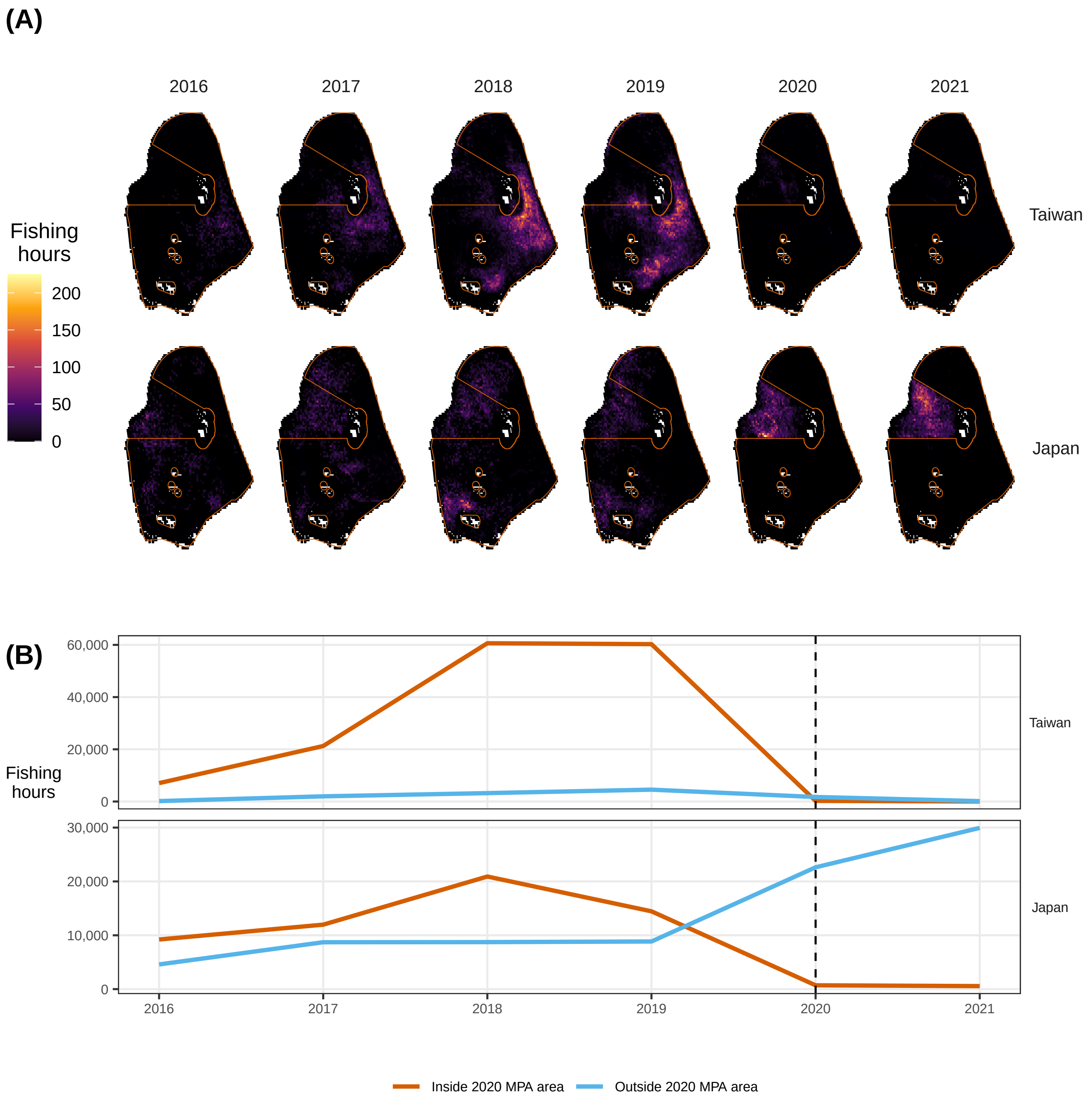

Fig. S13. Spatiotemporal trends of observed annual fishing effort from Global Fishing Watch in the Palau EEZ by Taiwanese and Japanese fleets from 2016 to 2021. (A) Spatial fishing patterns by flag and year, aggregated to 0.1x0.1 degree pixels; the outline of the Palau National Marine Sanctuary, which was implemented on January 1, 2020, is shown in dark orange. (B) Temporal trends of annual fishing effort, by flag, and disaggregated by inside and outside the Palau National Marine Sanctuary area, which was implemented on January 1, 2020 (a vertical dashed line is shown at this implementation date).

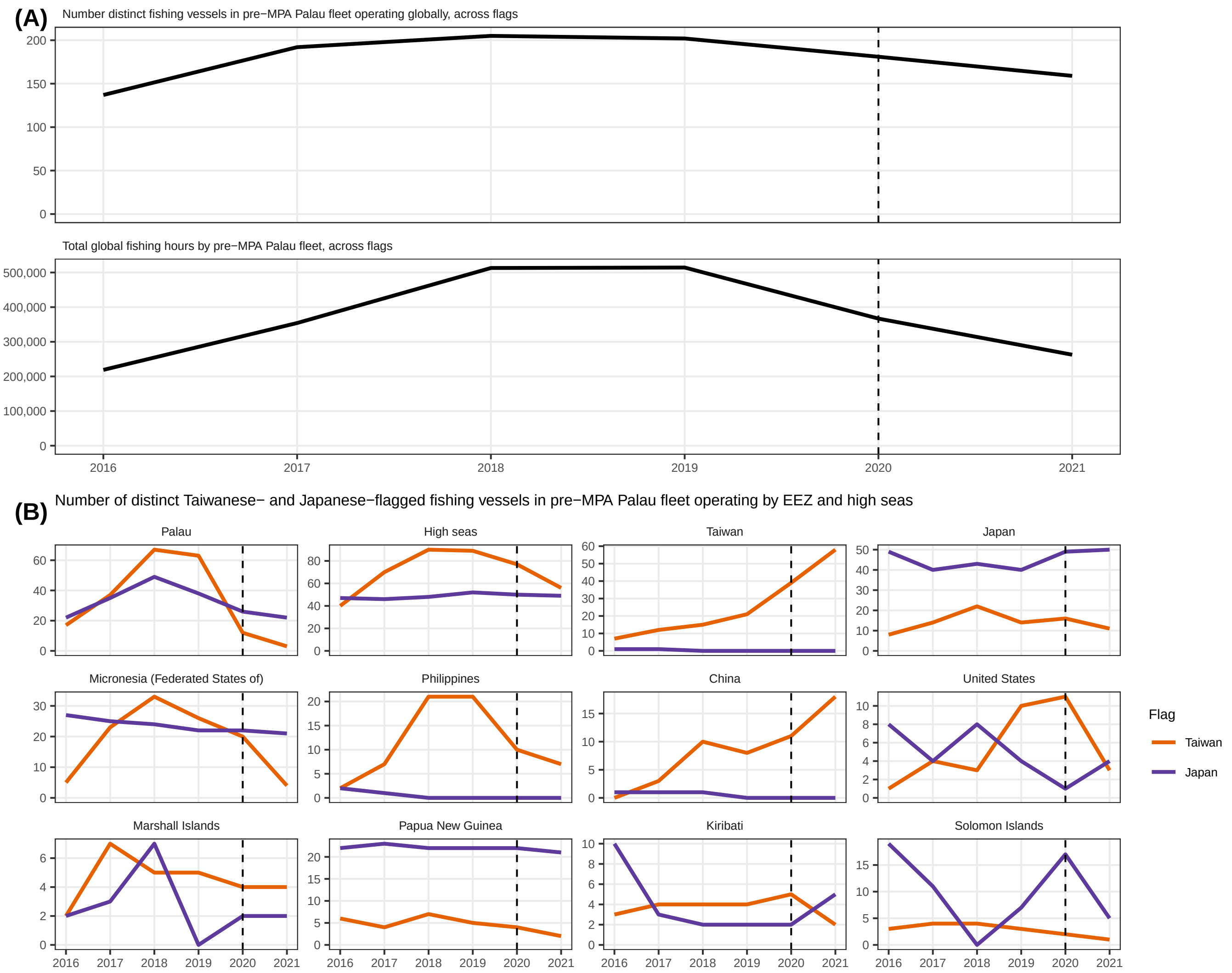

Fig. S14. Temporal trends of Palau's "pre-MPA fishing fleet" (i.e., those vessels observed fishing in Palau's EEZ between 2016 and 2019, prior to the Palau National Marine Sanctuary implementation on January 1, 2020). (A) shows the number of distinct vessels from this fleet that operated globally over time, as well as the total global fishing hours by this fleet over time. Panel A aggregates vessels and effort across all fishing flags. (B) shows the number of unique Taiwanese- and Japanese-flagged vessels from this fleet that fished in different EEZs and the high seas, focusing on the 12 regions with the largest number of active fishing vessels from this fleet. A vertical dashed line is shown at the January 1, 2020 implementation date of the Palau National Marine Sanctuary.

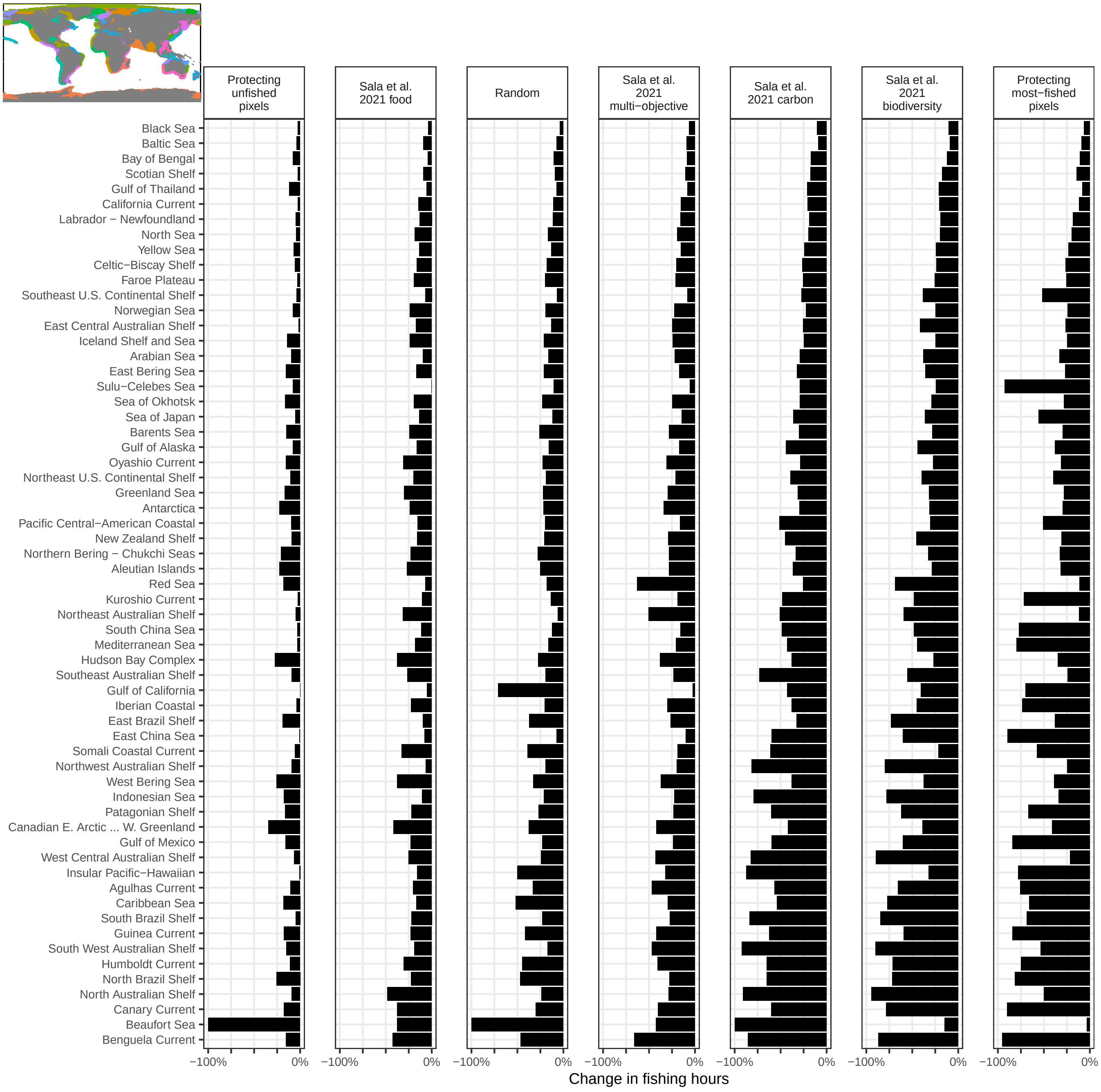

Fig. S15. Predicted change in fishing hours between various 30% MPA network scenarios and the business-as-usual counterfactual scenario, aggregated by Large Marine Ecosystem (LME). All results represent the predictions from a 3 year forecast horizon. An inset map of the LMEs is provided in the upper left corner.

References

1. K Sherman, AM Duda, Large marine ecosystems: an emerging paradigm for fishery sustainability. *Fisheries* **24**, 15–26 (1999).
